# Peripheral inflammation is associated with micro-structural and functional connectivity changes in depression-related brain networks

**DOI:** 10.1038/s41380-021-01272-1

**Published:** 2021-09-17

**Authors:** Manfred G. Kitzbichler, Athina R. Aruldass, Gareth J. Barker, Tobias C. Wood, Nicholas G. Dowell, Samuel A. Hurley, John McLean, Marta Correia, Charlotte Clarke, Linda Pointon, Jonathan Cavanagh, Phil Cowen, Carmine Pariante, Mara Cercignani, Neuroimmunology of Mood Disorders and Alzheimer’s Disease (NIMA) Consortium, Edward T. Bullmore, Neil A. Harrison

**Affiliations:** 1 University of Cambridge, Brain Mapping Unit, Department of Psychiatry, Downing Site, Cambridge, UK; 2 Institute of Psychiatry, Psychology and Neuroscience, Department of Psychological Medicine, King’s College London, London, UK; 3 University of Sussex, Brighton and Sussex Medical School, Clinical Imaging Sciences Centre, Brighton, UK; 4 University of Oxford Department of Psychiatry, Warneford Hospital, Oxford, UK; 5 University of Wisconsin, Department of Radiology, Madison, WI USA; 6 College of MVLS, Institute of Health and Wellbeing, University of Glasgow, Glasgow, UK; 7 MRC Cognition and Brain Sciences Unit, Cambridge, UK; 8 Centre for Immunobiology, University of Glasgow and Queen Elizabeth University Hospital, Glasgow, UK; 9 Cardiff University Brain Research Imaging Centre, Cardiff University, Cardiff, UK

**Keywords:** Depression, Neuroscience

## Abstract

Inflammation is associated with depressive symptoms and innate immune mechanisms are likely causal in some cases of major depression. Systemic inflammation also perturbs brain function and microstructure, though how these are related remains unclear. We recruited *N* = 46 healthy controls, and *N* = 83 depressed cases stratified by CRP (> 3 mg/L: *N* = 33; < 3 mg/L: *N* = 50). All completed clinical assessment, venous blood sampling for C-reactive protein (CRP) assay, and brain magnetic resonance imaging (MRI). Micro-structural MRI parameters including proton density (PD), a measure of tissue water content, were measured at 360 cortical and 16 subcortical regions. Resting-state fMRI time series were correlated to estimate functional connectivity between individual regions, as well as the sum of connectivity (weighted degree) of each region. Multiple tests for regional analysis were controlled by the false discovery rate (FDR = 5%). We found that CRP was significantly associated with PD in precuneus, posterior cingulate cortex (pC/pCC) and medial prefrontal cortex (mPFC); and with functional connectivity between pC/pCC, mPFC and hippocampus. Depression was associated with reduced weighted degree of pC/pCC, mPFC, and other nodes of the default mode network (DMN). Thus CRP-related increases in proton density—a plausible marker of extracellular oedema—and changes in functional connectivity were anatomically co-localised with DMN nodes that also demonstrated significantly reduced hubness in depression. We suggest that effects of peripheral inflammation on DMN node micro-structure and connectivity may mediate inflammatory effects on depression.

## Introduction

Depression and inflammation are strongly co-associated. Cohorts of patients with depression have increased blood concentrations of C-reactive protein (CRP) and pro-inflammatory cytokines compared to healthy controls. Patients with systemic inflammatory disease have increased incidence of “co-morbid” depressive symptoms [1, 2]. Longitudinal epidemiological studies [3–6], clinical studies of depression induced by interferon- treatment for hepatitis [7], and experimental studies of animal models of depression and sickness behaviour [8], collectively provide evidence that inflammation can precede and could cause depression.

Some of the clearest evidence that peripheral inflammation can cause changes in the human brain has come from functional magnetic resonance imaging (fMRI) studies of emotional or cognitive task-related activation. Experimental studies have collected fMRI data before and after controlled administration of a safe inflammatory challenge, e.g., typhoid vaccination of healthy volunteers, so that any before-after differences in brain function can be considered causal effects of peripheral inflammation [9]. Observational studies [10, 11] have reported correlations between task-related activation and experimentally uncontrolled between-subject variation in CRP or other peripheral immune biomarkers. Meta-analysis of 24 experimental and observational studies (total N~457) demonstrated replicable and significant effects of inflammation on task-related activation in dorsal anterior cingulate cortex (dACC), subgenual anterior cingulate cortex (sgACC) and adjacent areas of medial prefrontal cortex (mPFC), insula, hippocampus, amygdala, and striatum [12].

Functional MRI has also been used to investigate the relationship between peripheral inflammation and functional connectivity, usually measured by the correlation between resting-state fMRI time series at a pair of brain regions. Seed-based correlational analyses, typically focused on a few a priori regions of interest and discrete features of depression, have shown that typhoid vaccination-induced increases in interleukin-6 (IL6, a pro-inflammatory cytokine) were negatively correlated with mood-associated changes in functional connectivity of the sgACC in healthy volunteers [9]. In depressed patients, CRP has been shown to negatively correlate with the functional connectivity of both the striatum and amygdala to mPFC, which mediate specific depressive features of anhedonia and anxiety, respectively [13, 14]. More recently, studies using whole brain (connectomic) analysis have reported IL6-related changes in connectivity of sgACC and mPFC [15], lipopolysaccharide-induced (LPS) increases in cortico-subcortical connectivity [16], IFN-induced decreases in cortico-subcortical connectivity[17], and an internally replicated association between peripheral inflammation (indexed by a composite measure of CRP, IL6, and other cytokines) and reduced connectivity of an emotion regulation network [18]. Finally, a functional MRI sub-set of the data presented here was analysed using a complementary, whole-brain network based approach, which also showed widespread connectivity reduction with increased CRP [19]. However, task-related or resting-state fMRI is fundamentally limited as a marker of inflammation-related brain changes due to its lack of cellular or molecular specificity.

Micro-structural MRI is a complementary, though less-commonly used, approach to imaging effects of inflammation on the human brain. In contrast to more traditional macro-structural parameters, e.g., cortical thickness, which combine data from multiple voxels to measure brain anatomy, micro-structural MRI parameters represent the composition of brain tissue within each voxel. For example, magnetization transfer (MT) is a micro-structural MRI technique for measuring parameters which are biophysically interpretable in terms of the relative proportions of bound versus free water protons, or absolute proton density (PD). Experimental inflammatory challenges in healthy volunteers have induced significant changes in MT parameters in the striatum and other structures [20–22]. Clinical studies of patients with brain disorders with a known neuroinflammatory component, such as multiple sclerosis and stroke, have reported significantly increased PD, which has been interpreted as a marker of increased extracellular fluid volume, i.e., oedema, of brain tissue [23, 24].

Here we combined whole brain fMRI measurements of functional connectivity with MT measurements of micro-structure in an effort to elucidate how inflammation-related differences in the local, biophysical properties of brain tissue could be related to more distributed changes in functional connectivity between cortical and subcortical nodes of depression-related brain networks. CRP was used to stratify cases prospectively because it is measurable by a reliable, high sensitivity assay with a well-defined cut-off value for defining low grade systemic inflammation. Functional connectivity and micro-structural parameters were measured in the same set of 360 cortical areas and 16 subcortical regions in a sample of depressed cases (*N* = 83, including 33 with CRP > 3 mg/L) and healthy controls (*N* = 46). On this basis, we tested three principal hypotheses: (i) there are inflammation-related differences in quantitative MT parameters of brain tissue composition; (ii) there are inflammation-related differences in functional connectivity of cortico-subcortical networks; and (iii) inflammation-related differences in micro-structure and functional connectivity are anatomically co-located with each other and with depression-related changes in functional connectivity of cortico-subcortical networks.

## Methods and materials

### Study design and sample

Depression cases were ascertained as those participants who screened positive for current depressive symptoms on the Structured Clinical Interview for DSM-5 (SCID) screening questionnaire [25], scored >13 on the Hamilton Rating Scale for Depression [HAM-D]; [26] on two occasions (once at enrolment and again immediately prior to scanning), and screened negative for bipolar disorder or non-affective psychosis. The study was designed to be inclusive of some aspects of heterogeneity, including treatment resistance. Healthy controls screened negative for past or current depressive disorder on the SCID screening questionnaire. All participants satisfied additional inclusion and exclusion criteria (listed completely in Supplementary Information (SI) Table S1). In particular, to minimise the potentially cofounding effects of any systemic disorders that were likely to compromise the interpretation of immunological data, major medical disorders including type I and type II diabetes, severe cardiovascular disorders e.g., stroke, MI, and BMI ≥ 36 kg/m^2^, were all exclusionary. All procedures were approved by an independent national research ethics service (NRES) committee (NRES: East of England, Cambridge Central, UK; Reference: 15/EE/0092). All participants provided written informed consent and received up to £325 reimbursement.

We collected complete data from 143 eligible participants who were recruited into three a-priori groups: healthy controls with CRP < 3 mg/L (HC, *N* = 53), depressed cases with CRP < 3 mg/L (loCRP cases, *N* = 55), and depressed cases with CRP > 3 mg/L (hiCRP cases, *N* = 35). All groups were matched for mean age, sex and handedness. Subject to quality control criteria applied to MRI and other data, the final, evaluable dataset is summarised in Results, Table 1 and SI Fig. S1.

**Table 1:** Socio-demographic and clinical data on the analysable sample from this case-control study of depression cases, stratified by high or low CRP, and healthy controls.

	Controls	Cases	*p*	loCRP Cases	hiCRP Cases	*p*
*n*	46	83		50	33	
Sex (female/male)	27/19	57/26	0.335	29/21	28/5	0.015
Age, years	35.5 (7.5)	37.1 (7.3)	0.244	36.8 (7.1)	37.6 (7.6)	0.631
Body mass index	24.5 (4.2)	26.8 (4.1)	0.002	25.6 (3.7)	28.8 (3.9)	<0.001
C-reactive protein, mg/L	0.9 (0.7)	2.9 (2.9)	<0.001	1.0 (0.7)	5.8 (2.6)	<0.001
Clinician-rated depression^a^	0.5 (0.9)	19.3 (5.2)	<0.001	19.4 (5.4)	19.2 (4.9)	0.846
Self-rated depression^b^	2.0 (2.8)	25.3 (8.8)	<0.001	24.7 (8.4)	26.2 (9.4)	0.445
State anxiety^c^	26.9 (7.8)	51.2 (10.7)	<0.001	51.2 (10.8)	51.1 (10.7)	0.957
Trait anxiety	29.4 (5.9)	60.6 (9.2)	<0.001	60.3 (9.7)	60.9 (8.6)	0.767
Fatigue^d^	10.7 (2.5)	20.5 (5.7)	<0.001	20.4 (5.6)	20.6 (5.8)	0.866
Anhedonia^e^	0.2 (0.6)	5.0 (3.6)	<0.001	5.3 (3.4)	4.7 (3.8)	0.484
Childhood trauma^f^	38.2 (5.1)	53.2 (14.2)	<0.001	55.8 (14.9)	49.2 (12.3)	0.039
Treatment resistant	–	50	–	28 (56%)	22 (67%)	0.367
Number of antidepressants	–	2.7 (1.7)	–	2.6 (1.5)	2.9 (2.0)	0.405

^a^HAMD-17: Hamilton Rating Scale for Depression [51], a 17-item clinician-administered rating scale for reliable assessment of symptoms in patients diagnosed with depression, with a total score range 0–52.

^b^BDI: Beck Depression Inventory version 2 [52], a 21-item self-report questionnaire, with a total score range 0–63.

^c^STAI: Spielberger State-Trait Anxiety Rating Scale [53], a 40-item self-report measure of trait and state anxiety.[stai]

^d^CFS: Chalder Fatigue Scale [54], a 14-item self-report instrument to measure the severity of fatigue in adults.

^e^SHAPS: Snaith-Hamilton Pleasure Scale [55], a 14-item self-report measure of anhedonia (loss of the normal capacity for pleasurable experience), one of two principal symptoms of depression.

^f^CTQ: Childhood Trauma Questionnaire [56], a standardized, retrospective 28-item self-report inventory that measures the severity of five classes of childhood trauma: emotional abuse, physical abuse, sexual abuse, emotional neglect, and physical neglect.

#### Structural MRI data acquisition and pre-processing

Quantitative magnetization transfer (qMT) images were acquired using a magnetization transfer-weighted spoiled gradient echo sequence (voxel size 2.4 × 2.4 × 2.5 mm). The data were realigned and the following parameters estimated per voxel: proton density (PD), bound proton fraction (f_b_), MT exchange rate (k_bf_), and the transverse relaxation times of the bound and free water components (T2_b_, T2_f_). In order to account for differences in overall scanner sensitivity, we divided regional PD values by mean PD per subject, resulting in PD measurements globally normalized to unity. These maps were then regionally parcellated into 360 cortical regions, defined a priori using a well-validated parcellation template [27], and 8 subcortical regions defined bilaterally by the FreeSurfer atlas [28, 29]: thalamus, caudate, putamen, pallidum, hippocampus, amygdala, accumbens, and ventral diencephalon. This resulted in a 376-length vector for each of 5 regional qMT parameters for each participant.

### fMRI data acquisition and pre-processing

We used a multi-echo echoplanar imaging (EPI) sequence [30] to collect resting state fMRI data with the following parameters: TR = 2.57 s; total acquisition time = 10 mins 42.5 s = 250 time points; voxel size: 3.75 mm × 3.75 mm × 3.99 mm (for one site these parameters were marginally different, see SI page 4)

The first 6 volume were disregarded (for magnetization equilibration) then data were pre-processed using multi-echo independent component analysis (ME-ICA; [31, 32]) to identify sources of variance in the fMRI time series that scaled linearly with TE and could thus be confidently regarded as BOLD signal. Other non-BOLD sources of variance, such as head movement, that do not scale with TE, were identified by ME-ICA and discarded. The retained independent components yielded a denoised fMRI time series at each voxel which was bandpass filtered using the Maximal Overlap Discrete Wavelet Transform resulting in a BOLD signal oscillating in the frequency range 0.01–0.1 Hz (wavelet scales 2–4). For each participant, mean framewise displacement (FD) was calculated by averaging the FD time series. Scans for three participants were excluded due to high in-scanner motion, defined by prior quality control criteria of <FD>_RMS_ > 0.3 mm or max(FD) > 1.3 mm, and one other scan was excluded due to high mean correlation (r > 0.7) between all regional fMRI time series, which was considered to reflect systemic imaging artefacts.

Each pre-processed fMRI image was regionally parcellated into the same set of cortical and subcortical regions as the qMT data and the regional mean fMRI time series estimated for each cortical and sub-cortical region. Thus we estimated a 376 × 244 regional time series matrix for each participant. The functional connectivity between each regional pair of fMRI time series was estimated by Pearson’s correlation coefficient r for each possible pair of regions, resulting in a 376 × 376 symmetric association or functional connectivity matrix. Weighted degree of each node was calculated as the corresponding row (or column) mean of this matrix [33]. This sample is similar, but not identical, to the fMRI-only sample drawn from the same study for complementary analysis of functional connectivity [19].

### Statistical analysis

We adopted a hierarchical approach, proceeding from global through regional (nodal) to edge-wise scales of analysis. First, we used Kolmogorov-Smirnov tests to assess between-group differences in whole brain distributions of regional microstructural measures and functional connectivity (edge weights between each pair of regions). Significant group differences at the whole brain level were then further investigated for all 376 brain regions, with linear associations between CRP and microstructural measures or weighted degree estimated by regression for each regional node. Finally, linear association between CRP and functional connectivity was estimated by regression for each of 70,500 edges in the whole brain connectome and for each of the 375 edges connecting each subcortical structure to all other nodes. All mass univariate significance tests of between-group differences in MRI metrics, and associations between CRP and MRI metrics, were controlled for multiple comparisons by the false discovery rate (*P*_FDR_ < 0.05).

## Results

### Sample

Socio-demographic and clinical data are summarised in Table 1 for the sample with analysable fMRI data. Healthy controls were group mean-matched to all depressed cases on age and sex. As anticipated, there were significant case-control differences in depression, anxiety, fatigue, and anhedonia scores, as well as retrospectively self-reported childhood adversity exposure, with mean HAM-D scores falling in the moderate severity range. Depressed cases with high CRP included a higher proportion of women (85%) and had higher BMI (29) than cases with low CRP (58% female, BMI = 26). There were no significant differences between high and low CRP subgroups of cases in terms of clinician- or self-rated depression severity, state or trait anxiety, fatigue or anhedonia scores. Likewise, Within the group of cases, there were no significant correlations between CRP and any of these questionnaire measures of depressive psychopathology; see Fig. S15.

MT scans were excluded from one assessment centre (KCL) because of technical incompatibility with MT data from the other two centres (Oxford and Cambridge; see Fig. S14). Consequently, the sample size for micro-structural analysis is smaller (Table S2) than for fMRI analysis Table 1.

### Proton density: between-group differences and correlation with CRP

**Fig. 1:**
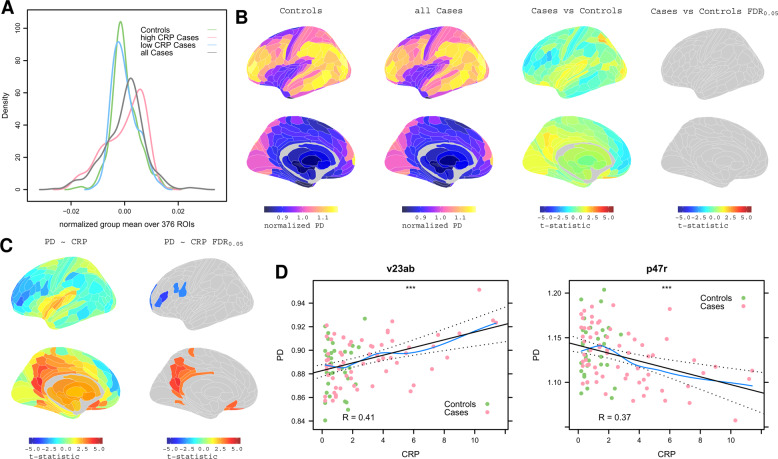
Proton density MRI: association with CRP. **A** Densities of normalized group mean PD over 376 regions for all cases, all controls, and for high CRP and low CRP subgroups of cases. **B** From left to right, cortical maps of: PD for all controls, PD for all cases, case-control difference in PD (t -statistic), and no significant case-control differences in PD with *P*_FDR_ < 0.05. **C** Left, cortical t -statistic map of association between regional PD and blood CRP concentration, PD~CRP, in all cases and controls combined; right, map of regions where PD~CRP was significantly greater than zero (orange regions), or less than zero (blue regions), with regional *P*_FDR_ < 0.05. **D** Left, scatterplot of PD (y-axis) versus CRP (x-axis) for posterior cingulate cortical region v23ab; right, scatterplot of PD versus CRP for dorsolateral prefrontal cortical region, p47r.

We estimated the global brain distribution of each MT parameter, across all 360 cortical and 16 sub-cortical regions for all groups. This demonstrated a highly significant difference in the distribution of PD (right-shifted) in high CRP (> 3 mg/L) versus low CRP (< 3 mg/L) depression cases (Kolmogorov-Smirnov test, *P* < 6.4 × 10^−7^) and between high CRP depresssed cases and controls (Fig. 1A). After controlling for multiple comparisons, there were no equivalent between-group differences for the global distributions of the other MT parameters (k_bf_, f_b_, T2_b_, T1_f_, T2_f_; see SI Fig. S2 and Table S3), so these were not further analysed.

Therefore, we next tested between-group differences in PD at each of 376 brain regions, controlling for multiple comparisons with *P*_FDR_ < 0.05. There were no significant differences between controls and all depressed cases (Fig. 1B). Taken together with the convergent distributional results in global PD, these regional data indicated that there was not a major effect of depression on PD. Therefore, we proceeded to investigate the association between PD and CRP using data from all participants (cases and controls combined).

We regressed PD on CRP at each region, resulting in a parcellated map of the association between PD and CRP, denoted PD~CRP (Fig. 1C, D). Proton density positively correlated with CRP in 22 regions with *P*_FDR_ < 0.05. This included 10 regions of posterior cingulate cortex and precuneus (pC/PC; RSC, PCV, 7 m, POS1, v23ab, d23ab, 31pv, 31pd, 31a, ProS), 6 regions of inferior, orbital and polar frontal cortex (IFJa, IFSa, a10p, p10p, p47r, OFC), 2 regions of anterior cingulate and medial prefrontal cortex (pOFC, 25), and single regions of dorsolateral prefrontal, premotor, paracentral and ventral visual stream cortex (9a, 6r, 5 m and VMV1, respectively). PD scaled negatively with CRP in 7 regions of prefrontal and premotor cortex. (See Table S4 for anatomical details).

### Functional connectivity: depression-related differences

**Fig. 2:**
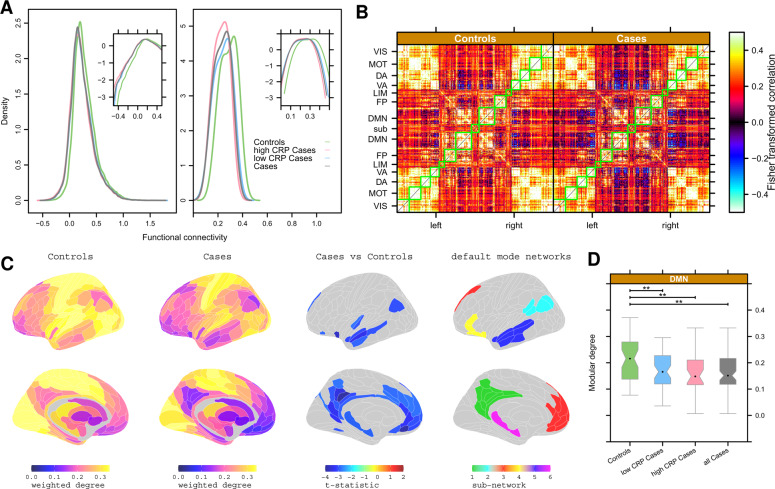
Functional connectivity: depression-related differences. **A** Left, group mean distributions of pair-wise correlation or functional connectivity for all controls, all cases, and high or low CRP subgroups of cases. Right, group mean distribution of weighted degree or nodal hubness for all controls, all cases, and high or low CRP subgroups of cases. Insets show the same data on a log scale to clarify between-group differences in the negative (or less positive) tails of the distributions. **B** Group mean functional connectivity matrices for all controls (left) and all cases (right). Each row or column in these symmetrical matrices represents one of 360 cortical regions which have been ordered by their prior affiliation to resting state networks or modules [57]: VIS = visual, MOT = motor, DA = dorsal attentional, VA = ventral attentional, LIM = limbic, FP = fronto-parietal, DMN = default mode network. In total, 16 subcortical regions are designated SUB. **C** Cortical and subcortical maps of group mean weighted degree for controls and cases, and the map of significant case-control differences in weighted degree (*P*_FDR_ < 0.05). In both groups, high degree hub nodes were concentrated in somatosensorimotor, visual and auditory cortices; low degree non-hub nodes were concentrated in DMN cortical areas and subcortical structures. There was significantly reduced hubness of nodes in many cortical areas of the default mode network (DMN, right column). **D** Boxplots of weighted degree of all cortical nodes in the DMN for all controls, all cases, and high or low CRP subgroups of cases. ** denotes significant between-group differences in modular degree of DMN (*P* < 0.05).

We plotted the distributions of all pair-wise fMRI time series correlations (70,500) on average over all participants in each (sub)group (Fig. 2A, left). Distributions were generally positive, but differed significantly between cases and controls (Kolmogorov-Smirnov [KS] test, *P* < 2.2 × 10^−16^). Depressed cases had a functional connectivity distribution left-shifted compared to controls, indicating a greater proportion of negatively weighted functional connections, especially in the high CRP subgroup.

We also plotted the distribution of all nodal measures of weighted degree (i.e., connectivity of each region to all other regions) on average over all participants in each (sub)group (Fig. 2A, right). Weighted degree was always positive in the range 0.07–0.42 but, in depressed cases, the degree distribution was significantly left-shifted, indicating fewer hub nodes with high degree and more non-hub nodes with near-zero degree (KS, *P* < 2.2 × 10^–16^). Within the group of depressed cases, the degree distribution was left-shifted in high CRP versus low CRP subgroups (KS, *P* < 5 × 10^−5^).

To localise these case-control differences in functional connectivity, we estimated the mean inter-areal correlation matrix for each group Fig. 2B. These functional connectomes had a block-diagonal appearance, representing the modular community structure of the networks, for both groups. Regions in the same modules were generally positively correlated with each other. In contrast, there were strong negative correlations between the default mode network (DMN) and the ventral attention (VA) and dorsal attention (DA) modules, especially in depressed cases compared to controls (see Fig. S7 for an alternative approach using ICA components instead of the pre-defined Yeo networks).

We used weighted degree as a measure of hubness of cortical node in the functional connectome (Fig. 2C). Depression was associated with significantly reduced hubness (i.e., global connectivity) of 39 regions of pC/pCC, inferior parietal cortex, mPFC and hippocampus (*P*_FDR_ < 0.05; Table S4), many of which were affiliated to the DMN (Fig. 2C). More formally, the mean degree of all nodes within the DMN was significantly reduced for depressed cases compared to controls; and there were no significant case-control differences of modular degree for any other prior modules (Fig. 2D, Fig. S3).

### Functional connectivity: correlation with CRP and mediation analysis

In light of the significant depression-related differences in functional connectivity, we next estimated the association between CRP and functional connectivity using data from all depressed cases (excluding controls).

**Fig. 3:**
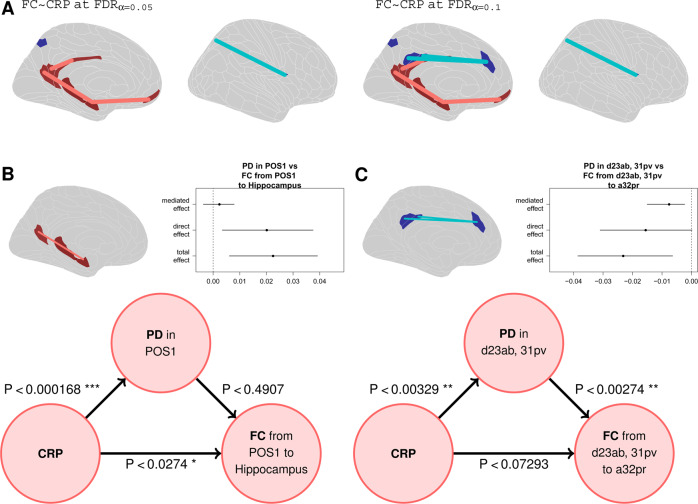
Functional connectivity: correlation with CRP and mediation analysis. **A** Cortical maps highlighting edges between regional nodes defined by significant correlation of functional connectivity with CRP (FDR corrected at 0.05 and 0.1). **B** Mediation analysis of the direct effect of CRP on PD in posterior cingulate cortical area POS1, the direct effect of CRP on functional connectivity between POS1 and hippocampus (represented by the red edge between nodes in the brain map), and the effect of PD on POS1-hippocampal connectivity. The direct effects of CRP on POS1 PD and hippocampal connectivity are significant but there is no indirect effect of CRP on hippocampal connectivity mediated by its effect on PD in posterior cingulate cortex. **C** Mediation analysis of the direct effect of CRP on PD in posterior cingulate cortical areas d23ab and 31pv, the direct effect of CRP on functional connectivity between d23ab/31pv and medial frontal area a32pr (represented by the blue edge between nodes in the brain map), and the effect of PD on d23ab/31pv-hippocampal connectivity. The direct effect of CRP on d23ab/31pv PD and there is an indirect effect of CRP on medial prefrontal connectivity mediated by its effect on PD in posterior cingulate cortex. (ACME: average causal mediation effect, ADE: average direct effect).

Following our hierarchical analysis plan, we finally regressed CRP on each of 70,500 pair-wise inter-regional correlations to assess inflammation-related changes in functional connectivity at the most fine-grained scale of the connectome, i.e., individual edges rather than regional nodal degrees or global correlation distributions. Three regional pair-wise connections scaled positively with CRP (*P*_FDR_ < 0.05), two between DMN nodes (pc/pCC areas: v23ab to RSC, and POS1 to hippocampus), and one between the hippocampus and mPFC (area 10r). One connection scaled negatively with CRP, between area 7Pm (adjacent to pC/pCC) and the frontal-opercular area, FOP1 (Fig. 3A). At more lenient FDR thresholds more negative edges started to appear. Specifically at *P*_FDR_ < 0.1 we see two connections between the PCC (areas d23ab and 31pv) and the dorsal ACC (area a32pr) whose strength scales negatively with CRP.

**Fig. 4:**
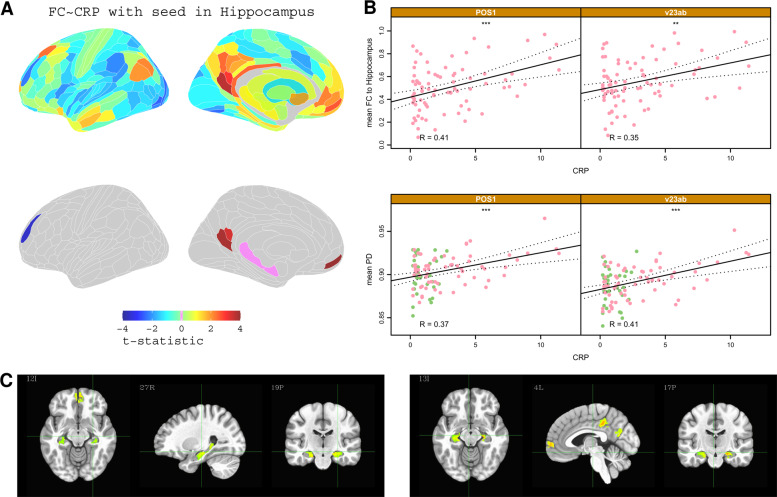
Hippocampal connectivity: correlation with CRP. **A** Top row, brain maps of t-statistics representing strength of association between CRP and functional connectivity of the hippocampus (with each of the other brain regions). Bottom row, cortical maps highlighting regions where CRP was significantly, positively correlated with functional connectivity with the hippocampus (*P*_FDR_ < 0.05). **B** Scatterplots illustrating the relationship between CRP and hippocampus connectivity for two cortical regions: POS1 and v23ab. Bottom, scatterplots of CRP versus PD for the same cortical regions; * denotes *P* < 0.05. **C** Voxel based analysis of functional connectivity dependence on CRP for a seed in the PCC (top row) and for a seed in the right Hippocampus (bottom row). Functional connectivity between hippocampus, PCC, and mPFC was positively correlated with CRP (only significant clusters with *α* < 0.05 are shown; global significance threshold *P* < 0.05).

Given the involvement of the hippocampus in two of the three connections that positively scaled with CRP, we used seed-based correlational analysis to investigate the association between CRP and the functional connectivity of the hippocampus with each of the other 187 regions per hemisphere. This showed that high CRP was associated with increased functional connectivity between the hippocampus and 3 other regions located in the pC/pCC and mPFC (Fig. 4A, C; see Table S5 for details of CRP-related changes in connectivity of hippocampus, putamen and thalamus). We explored the associations between functional connectivity of each of these edges and participants’ psychopathology scores including anxiety and anhedonia; however, none of these associations were statistically significant after controlling for multiple comparisons, see Fig. S16 for details.

Notably, some of the cortical areas, e.g., POS1, v23ab, where functional connectivity scaled significantly with CRP also demonstrated a significant association between PD and CRP (Fig 4B; but not depression, cf. Figures S10–11). This co-localisation (Fig. S8) of inflammation-related effects on brain micro-structure and functional connectivity could represent either a direct effect of CRP on both PD and functional connectivity, or an indirect (mediated) effect of CRP on functional connectivity via its direct effect on PD (or vice-versa). We used mediation analysis to evaluate these models for two specific examples of structural/functional co-localisation of CRP-related effects: (1) CRP scaled positively with PD in the pC/pCC area POS1, and scaled positively with functional connectivity between POS1 and hippocampus. Here, mediation analysis indicated a direct effect of CRP on hippocampal connectivity. (2) CRP scaled positively with PD in pc/PCC areas d23ab and 31pv, and scaled negatively with functional connectivity between d23ab/31pv and mPFC area a32pr. Here, mediation analysis indicated that the association of CRP with medial prefrontal connectivity was mediated by its direct effect on PD in posterior cingulate cortex.

## Discussion

We tested the prior hypotheses that peripheral inflammation is associated with disruptions in human brain micro-structure and functional connectivity that co-locate with depression-related changes in functional networks. Our results supported these predictions and generated new hypotheses about how peripheral inflammation may perturb brain structure and function, and their role in depression.

### Inflammation-related changes in proton density

The clearest signal of inflammation-related change was provided by the micro-structural MRI measurements of proton density (PD) at global (Fig. 1A) and regional scales of analysis (Fig. 1C). We found 22 areas of association cortex, particularly within the DMN, where PD scaled positively (pC/PCC and mPFC) or to a lesser extent negatively (DLPFC) with CRP. These results were robust to multiple comparisons correction at P_FDR_ < 0.05 and to sensitivity analyses including correction for potentially confounding variables (sex, BMI, childhood adversity CTQ, total number of antidepressants, whether currently on antidepressants, as well as Global Signal Regression) and restriction to data from depressed cases only (Fig. S4).

Proton density measures the concentration of MRI-visible protons. In the brain, most protons are in water, and PD is typically interpreted as a measure of tissue-free water content [34]. In clinical studies of stroke, glioma, multiple sclerosis (MS) and hepatic encephalopathy [23, 24, 35, 36] increased PD has typically been interpreted as a marker of increased extracellular fluid volume, or oedema [35, 37]. Convergently, animal experiments have shown that peripheral inflammation can cause increased free water in brain tissue measured directly by a gravimetric technique [38].

Thus, an intuitive interpretation of our finding that CRP was associated with increased PD in pC/PCC and mPFC is that low-grade systemic inflammation is associated with localised oedema of these components of the DMN. This accords with studies reporting acute reductions in brain volume (interpreted as a reduction in hydration and extracellular fluid volume) after initiation of potent immunosuppressive therapies in multiple sclerosis, the so-called “pseudoatrophy phenomenon” [39]. However, analogous to our finding of regions showing a negative scaling of PD with CRP, some prior studies have also reported regional increases in brain volume (interpreted as an increase in hydration and extracellular fluid volume) following anti-inflammatory therapies [40]. Thus there is prior evidence, compatible with our findings, that variation in systemic inflammation can cause both localised increases and decreases in the water composition of brain tissue. Further studies, ideally translating between MRI studies of animals and humans, will be needed to validate the biophysical interpretation of inflammation-related changes in PD, to clarify the regional specificity of brain water associations with peripheral pro- or anti-inflammatory states [40], and to understand why PD is apparently more sensitive to the effects of systemic inflammation on brain tissue composition than other micro-structural MRI markers of free water content, such as MT or T1 [41].

### Depression-related changes in functional connectivity

The clearest signal of depression-related change was provided by fMRI measurements of functional connectivity, at global (Fig. 2A, B) and regional scales of analysis (Fig. 2C, Table S4). We found that 39 cortical areas and the hippocampus had significantly attenuated hubness, or degree of connectivity to the rest of the connectome, of which 27 (69%) were affiliated to the DMN. These results were robust to several sensitivity analyses including correction for BMI, head movement, and other potentially confounding variables (Fig. S4).

Depression-related abnormalities of task-related activation and functional connectivity of sgACC, pC/PCC and other nodes of the DMN have been extensively reported [42–44]. Increased connectivity between DMN nodes, but decreased connectivity between DMN and non-DMN nodes, has been conceived as an imbalance between task-negative (DMN) and task-positive (non-DMN) systems that underpins cognitive biases in depression [45, 46]. Meta-analyses have highlighted the importance of dysconnectivity between the pC/pCC and the ventral attention and frontoparietal networks for the emergence of ruminative thoughts and emotional dysregulation characteristic of depression [47]. Our finding of reduced degree of functional connectivity of 39 DMN areas is compatible with depression-related disconnection of the DMN from wider task positive areas.

### Mechanistic interpretation

One pathogenic hypothesis generated by these data is that the posterior DMN is particularly susceptible to localised increase in extracellular water, induced by peripheral inflammation, and indexed by PD. We used CRP as a convenient, well-established proxy for peripheral innate immune system activation; but we don’t assume that CRP itself traverses or signals across the blood brain barrier (BBB). Instead, it seems more likely that high CRP serves as a surrogate for increased blood concentrations of pro-inflammatory cytokines or myeloid immune cells which can traverse the BBB to exert effects on inflammation-responsive brain regions [48]. Whatever the precise mechanism of peripheral immune signalling to the pC/pCC, this region is central to the regulation of information flow throughout the DMN, via connections to the hippocampus and mPFC [44]. Thus inflammation-induced oedema of pC/PCC could perturb the functional connectivity between nodes within the DMN, and/or between DMN and non-DMN nodes, ultimately causing changes in emotion and cognition diagnosed clinically as depression [45]. We found preliminary evidence that localised inflammatory effects on posterior cingulate cortex micro-structure could mediate indirect effects of inflammation on some functional connections of the putatively edematous areas. However, causality cannot be inferred from our current cross-sectional study and further translational and longitudinal MRI studies will clearly be needed to investigate this mechanistic model linking inflammatory effects on cortical micro-structure to dysconnectivity of depression-related functional networks.

### Methodological issues and limitations

The sample size is not large so there is a risk of inadequate power to detect small effects of depression or inflammation in sub-group analyses of the control group alone (see Figs. S12 and S13). Future studies of non-depressed control groups with unrestricted variation in CRP, e.g., the UK Biobank sample, will be required to clarify the relationship between functional connectivity and high CRP in the absence of depressive symptoms. Relatedly, serious medical disorders (including type I and type II diabetes, severe cardiovascular disorders and BMI ≥ 36 kg/m^2^) were exclusionary to avoid compromising interpretation of immunological data. It will therefore be important to determine the generalizability of our findings to depressed populations with higher levels of obesity or medical co-morbidity. The depressed cases (but not controls) were enriched for patients with CRP > 3 mg/L, potentially confounding depression- and inflammation-related effects on MRI markers. However, this risk was mitigated by robustness of key results to sensitivity analyses of data from cases only. In the principal analysis, we controlled for age and scanning centre; but not for effects of sex, BMI and childhood adversity, given prior evidence that all of these factors are risk factors for both depression and inflammation. However, key results were conserved after statistical correction for BMI and childhood adversity, and analysis of female-only data (Figs. S5 and S6). Sensitivity analyses controlling for anti-depressant drug treatment-resistance included as a covariate the total number of previous episodes of anti-depressant treatment with different drugs (Table 1, Fig. S4). Head movement during scanning is a well-recognised source of bias in fMRI connectivity analysis. We used a well-validated pre-processing pipeline for movement correction and all data passed standard quality control criteria for head movement before statistical analysis (see Fig. S9). Nonetheless, we used global signal regression [49] as an alternative pre-processing strategy for motion correction in sensitivity analyses that demonstrated conservation of our key results (Fig. S4).

Finally, a number of studies have demonstrated scaling of CRP and/or IL6 with specific phenotypic dimensions of depression (such as anhedonia, psychomotor slowing, or anxiety). Some of these dimensions of depressive psychopathology have also been linked to inflammation-related changes in mPFC connectivity to ventral and dorsal striatum, and amygdala [13, 14, 50]. It is therefore noteworthy that we also observed significant CRP-related changes in mPFC functional connectivity. However, we did not observe significant associations between CRP and scores on questionnaire measures of depressive symptom severity, anxiety, anhedonia or fatigue in this group of depressed cases (Fig. S15). Perhaps unsurprisingly in this context, there was also no evidence for significant association between any of these questionnaire scores and the strength of functional connections that were significantly correlated with CRP (Fig. S16). Conceptually, it seems likely that inflammation-related changes in functional connectivity should be related to specific dimensions of depressive psychopathology. More refined investigation of this concept is warranted as an important focus for future fMRI connectivity studies using larger samples of depressed cases with more comprehensive phenotyping of both psychopathological or cognitive phenotypes and peripheral immune profiles.

## Supplementary information

Supplementary Information

## Data Availability

The analysis packages used in this study are freely available through the following links:Structural analysis and registration: https://freesurfer.net Micro-structural analysis of qMT data: https://github.com/spinicist/QUIT Functional analysis of resting state multi-echo fMRI data: https://github.com/manfredg/MEICA-BMU.
