# Supplementary material for: Peripheral inflammation is associated with micro-structural and functional connectivity changes in depression-related brain networks
Source: Mol Psychiatry. 2021 Sep 17;26(12):7346–54. doi: 10.1038/s41380-021-01272-1 (PMC8872995; doi:10.1038/s41380-021-01272-1)
Supplement: Supplementary file 1 — Supplementary Information [file 41380_2021_1272_MOESM1_ESM.pdf]

Peripheral inflammation is associated with micro-structural and functional connectivity changes in depression-related brain networks

Supplementary Information

Manfred G Kitzbichler[a], Athina R Aruldass[a], Gareth J Barker[b], Tobias C Wood[b], Nicholas G Dowell[c], Samuel A Hurley[e,f], John McLean[g], Marta Correia[i], Charlotte Clarke[c], Linda Pointon[a], Jonathan Cavanagh[h], Phil Cowen[e], Carmine Pariante[b], Mara Cercignani[c], Mara Cercignani[c], Edward T Bullmore[☆a], Neil A Harrison[☆c,d,], on behalf of theNeuroimmunology of Mood Disorders and Alzheimer's Disease (NIMA) Consortium

[a] University of Cambridge, Brain Mapping Unit, Department of Psychiatry, Downing Site, Cambridge CB2 3EB, UK
[b] Institute of Psychiatry, Psychology and Neuroscience, Department of Psychological Medicine, King's College London, SE5 9RT, London, UK
[c] University of Sussex, Brighton and Sussex Medical School, Clinical Imaging Sciences Centre, Brighton BN1 9RR, UK
[d] Cardiff University Brain Research Imaging Centre, Cardiff University, Maindy Road, Cardiff, CF24 4HQ, UK
[e] University of Oxford Department of Psychiatry, Warneford Hospital, Oxford, OX3 7JX, UK
[f] University of Wisconsin, Department of Radiology, Madison, WI USA 53705-2275
[g] College of MVLS, Institute of Health and Wellbeing, University of Glasgow, Glasgow, G51 4TF, UK
[h] Centre for Immunobiology, University of Glasgow and Queen Elizabeth University Hospital, Glasgow, G51 4TF, UK
[i] MRC Cognition and Brain Sciences Unit, Cambridge CB2 7EF, UK

Contents

Methods and Materials in detail ***1***
- *Study design and sample* . . . 1
- *Structural MRI data acquisition* . . . 2
- *Functional MRI data acquisition* . . . 2

Clinical and Behavioral Assessment ***3***
- *Clinical evaluation* . . . 3
- *Clinical assessments* . . . 4
- *Medical comorbidities and concomitant medication* . . . 4

Members of the NIMA Consortium ***4***

Bibliography ***5***

Tables ***6***

Figures ***10***

Methods and Materials in detail

Study design and sample

This was an observational, multi-site, case-control study. Depressed cases screened positive for current depressive symptoms on the Structured Clinical Interview for DSM-5 screening questionnaire (SCID; First et al., 2016) and had total score greater than 13 on the Hamilton Rating Scale for Depression (HAM-D; Hamilton, 1960) on two occasions (once at enrolment and again immediately prior to scanning), and screened negative for bipolar disorder or non-affective psychosis. Healthy controls screened negative for past or current depressive disorder on the SCID screening questionnaire. All participants satisfied additional inclusion criteria, e.g. aged 25-50 years, and exclusion criteria, e.g. major medical inflammatory disorder or immuno-modulatory medication, as detailed in Supplementary Information (SI) **Table S1** and Medical comorbidities section below. Depressed cases were stratified by venous blood concentration of CRP: high CRP cases had CRP > 3 mg/L; low CRP cases had CRP < 3 mg/L. All controls had CRP < 3 mg/L.

After preliminary telephone screening, potentially eligible participants attended one of 5 UK recruitment centres (Brighton, Cambridge, Glasgow, King's College London (KCL), or Oxford) for clin-

ical interviews and blood sampling for CRP. Eligible participants then attended one of three UK assessment centres (Cambridge, KCL, Oxford) for venous blood sampling, clinical assessment, and a single MRI scanning session; see SI **Figure S1** for details. Between-site reliability of fMRI connectivity and MT parameters was established by a pilot study of 5 healthy volunteers, each scanned at all three assessment centres, confirming that the coefficient of variability (CoV) was lower between sites than between subjects.

We collected complete data from 143 eligible participants categorised into three groups: healthy controls (HC, N=53), depressed cases with CRP < 3 mg/L (loCRP cases, N=55), and depressed cases with CRP > 3 mg/L (hiCRP cases, N=35). All groups were matched for mean age, sex and handedness (Edinburgh Handedness Inventory). Subject to quality control criteria applied to MRI and other data, the final, evaluable dataset is summarised in Results, **Table 1** and SI **Figure S1**.

All procedures were approved by an independent national research ethics service (NRES) committee (NRES: East of England, Cambridge Central, UK; Reference: 15/EE/0092). All participants provided written informed consent and received up to £325 reimbursement.

Additional clinical assessments

All participants additionally completed the following self-report standardized instruments (see also footnotes **Table 1** and SI): Beck Depression Inventory version 2 (BDI-II), Snaith-Hamilton Pleasure Scale (SHAPS), State-Trait Anxiety Inventory (STAI), Chalder Fatigue Score (CFS), Childhood Trauma Questionnaire (CTQ), Perceived Stress Scale (PSS) and Life Events Questionnaire (LEQ).

Biomarker assessments

We collected 50 mL of venous blood at 8-10 a.m. on the day of assessment. Participants had fasted since 10 p.m. the previous night and had been lying supine for 30 mins prior to venepuncture. C-reactive protein was measured using a high sensitivity assay at a single central laboratory (Q^2 Solutions, The Alba Campus, Livingston EH54 7EG, UK) from 0.5 mL of plasma (reportable range 0.2-9999.9 mg/L).

Body mass index (BMI) was measured as weight (kg) divided by height squared (m^2).

Structural MRI data acquisition

Quantitative magnetization transfer (qMT) images were acquired using a magnetization transfer-weighted spoiled gradient echo sequence comprising a set of 10 combinations of flip angle (2: 360 and 720 deg) and off-resonance frequency (5: 1-25 kHz) with the following parameters: acquisition time = 20 min; relaxation time (TR) = 32 ms; echo time (TE) = 2.9 ms; field of view (FoV) 192 mm, equivalent to 64 axial slices at matrix size 80×80 and voxel size 2.4×2.4×2.5 mm.

Structural MRI data pre-processing

The qMT data were realigned to subject specific structural images using rigid-body registration; qMT parameters were then estimated by voxel-wise non-linear least squares fitting (Levenberg-Marquardt) of a binary spin bath model using the QUIT package (QUantitative Imaging Tools; Wood, 2018). This yielded whole-brain maps of proton density (PD), bound proton fraction (f_b), MT exchange rate (k_{bf}), and the transverse relaxation times of the bound and free water components ($T2_b$, $T2_f$). Spatial intensity variations (bias field, RF inhomogeneities) were corrected using FSL FAST (Zhang et al., 2001). In order to account for differences in overall scanner sensitivity, we divided regional PD values by mean PD per subject, resulting in PD measurements globally normalized to unity.

Quantitative MT parameter maps were then regionally parcellated into 360 cortical regions, defined *a priori* using a well-validated parcellation template (Glasser et al., 2016), and 8 subcortical regions defined bilaterally by the FreeSurfer atlas (Fischl, 2012; Fischl et al., 2002): thalamus, caudate, putamen, pallidum, hippocampus, amygdala, accumbens, and ventral diencephalon. This resulted in a 376-length vector for each of 5 regional qMT parameters for each participant.

Functional MRI data acquisition

We used a multi-echo echoplanar imaging (EPI) sequence (Poser et al., 2006) to collect fMRI data under resting state conditions with the following parameters: TR = 2.57 s; echo times ($TE_{1,2,3}$) = 15 ms, 34 ms and 54 ms; total acquisition time = 10 mins 42.5 s = 250 time points in each fMRI time series. Multi-echo EPI data were collected as 32 slices at -30 degrees downward pitch to the AC-PC line, with field of view 240 mm; matrix size 64×64; and voxel size: 3.75 mm×3.75 mm×3.99 mm. For one site these parameters were marginally different as described in the following paragraph.

Differences in fMRI acquisition parameters for the KCL site

The scans collected at the Kings College London used a multi-echo echoplanar imaging (EPI) sequence with the following parameters: relaxation time (TR) = 2.5 s; echo times ($TE_{1,2,3}$) = 15 ms, 34 ms and 54 ms; total acquisition time = 10 mins 25 s = 250 time points in each fMRI time series; collected as 35 slices at -30 degrees downward pitch to the AC-PC line, field of view: 240 mm, matrix size: 64×64, voxel size: 3.75 mm×3.75 mm×3 mm (with a 1 mm gap).

fMRI data pre-processing

The first 6 volumes were discarded to ensure scanner equilibrium and the remaining data were pre-processed using multi-echo independent component analysis (ME-ICA; Kundu et al., 2012, 2013) to identify sources of variance in the fMRI time series that scaled linearly with TE and could thus be confidently regarded as BOLD signal. Other non-BOLD sources of variance, such as head movement, that do not scale with TE, were identified by ME-ICA and discarded. The retained independent components, representing BOLD contrast, were optimally recomposed to generate a broadband denoised fMRI time series at each voxel. This was band-pass filtered using the Maximal Overlap Discrete Wavelet Transform ("modwt" using "la8", the Daubechies orthonormal compactly supported wavelet of length L=8), resulting in a BOLD signal oscillating in the frequency range 0.01-0.1 Hz (wavelet scales 2-4).

Geometric re-alignment was used to estimate 6 motion parameters for each participant (3 translation and 3 rotation parameters)

which were used to calculate an overall estimate of motion - frame-wise displacement (FD) - defined as the Euclidean norm of motion and rotation derivatives in mm: $FD^2 = |\vec{\Delta}x|^2 + |\vec{\Delta}\theta|^2$). For each participant, mean FD was calculated by averaging the FD time series. A total of three scans were excluded due to high in-scanner motion $\langle FD \rangle_{RMS} > 0.3$ mm or max(FD) > 1.3 mm and one subject was dropped due to excessively high mean correlation > 0.7.

Each pre-processed fMRI image was regionally parcellated into the same set of cortical and subcortical regions as the qMT data and the regional mean fMRI time series estimated for each cortical and sub-cortical region using the non-zero mean variant of the AFNI *3dROIstats* command (Cox, 1996). Thus we estimated a 376×244 regional time series matrix for each participant.

The functional connectivity between each regional pair of fMRI time series was estimated by Pearson's correlation coefficient *r* for each possible pair of regions, resulting in a 376×376 symmetric association or functional connectivity matrix. The row (or column) means of this matrix comprise the vector of regional or nodal weighted degree (Fornito et al., 2016).

Statistical analysis

We used Kolmogorov-Smirnov (KS) tests to assess between-group differences in whole brain distributions of regional PD, regional weighted degree, and functional connectivity or edge weight between each pair of regions. Between-group differences in PD and weighted degree were estimated and tested separately for all 376 brain regions. Likewise linear association between CRP and PD or weighted degree was estimated by regression for each regional node. Linear association between CRP and functional connectivity was estimated by regression for each of 70,500 edges in the whole brain connectome and for each of the 375 edges connecting each subcortical structure to all other nodes. All mass univariate significance tests of between-group differences in MRI metrics, or associations between CRP and MRI metrics, were controlled for multiple comparisons by the false discovery rate ($P_{FDR} < 0.05$) and KS tests by Bonferroni correction for the 30 different group comparisons (5 qMT parameters and 6 different pairs) investigated.

Clinical and Behavioral Assessment

Clinical evaluation

Age, gender, medical history, participant details and family history were documented using semi-structured clinical interview by trained non-clinician research assistants.

Structured Clinical Interview for *DSM-5* (SCID)

First et al. (2016). The 2-item screening of core depressive symptoms (low mood or depression and anhedonia) and assignment of depression diagnosis was operationalized using the SCID for *Diagnostic and Statistical Manual for Mental Disorders*, 5th. edition (DSM-5) Depressive Disorders screening questionnaire. The instrument was administered by trained non-clinician research assistants on two occasions, i.e. during initial screening visit, and again immediately prior to MRI (see **Figure S1**. All cases screened positive for current depressive symptoms on the Structured Clinical Interview for DSM-5 (SCID) screening questionnaire and scored >13 on the Hamilton Rating Scale for Depression (HAM-D) on two occasions (once at enrolment and again immediately prior to scanning), and screened negative for bipolar disorder or non-affective psychosis.

Hamilton Rating Scale for Depression (HAM-D)

Hamilton (1960). The HAM-D is a 21-item observer-rated instrument designed to measure severity of depression. Only the first 17 items are weighted towards severity measurement. The additional 4 items - assessing for diurnality in symptom presentation, paranoia and obsessive-compulsive behaviors - reflect the type of depression and presentation of rarely occurring symptoms, as opposed to measuring intensity of depression. Thus, the final 4 variables were excluded from the rating scale. Each item is assessed against a 5-point (0-4; 8 items) or 3-point (0-2; 9 items) scale, with reverse-scoring for item-17(Insight). Total scores range from 0-52, with greater scores indicating more severe depression. The questionnaire is extensively used and has high reliability and validity (Knesevich et al., 1977). In our study, the HAM-D was used alongside the SCID for DSM-5 screening questionnaire as part of the eligibility criteria (see **Table S1**) and to measure severity of current depressive state in participants.

Beck Depression Inventory version 2 (BDI-II)

Beck and Beamesderfer (1974). The 21-item self-reported questionnaire BDI-II was used to measure severity of disease. Akin to the HAM-D, the instrument indicates severity of depression through general assessment of somatic, affective and cognitive symptoms. Each item is scored against a 4-point scale (0-3), with greater total scores (ranging from 0 to 63) indicating greater severity.

Snaith-Hamilton Pleasure Scale (SHAPS)

Snaith et al. (1995). This 14-item self-reported instrument was used to measure hedonic tone or more strictly, anhedonia – one of the core symptoms of depression (Snaith et al., 1993). Each item has a 4-level Likert scale i.e. "Definitely agree", "Agree", "Disagree" and "Strongly disagree". The former two "Agree" responses receive a score of 1, whilst the latter two "Disagree" responses receive 0. Thus, total scores range from 0 to 14, with higher scores indicating higher levels of current anhedonia or reduced hedonic experience.

State-Trait Anxiety Inventory (STAI)

Spielberger (1983). The 40-item STAI was used to measure State-anxiety (STAI-S) and Trait-anxiety (STAI-T) with 20 items loading onto each factor (or subset) i.e. first 20 items for STAI-S and, subsequent 20 items for STAI-T. State-anxiety is a measure of the current state of anxiety i.e. severity of anxious symptom experienced by the subject. These are transient feelings. Trait-anxiety in contrast, is a persistent or consistent experience of anxiety, may be conceptualized as a personality trait and thus a risk factor for depression. Similar to the SHAPS, each item within each subset have 4-category response. Scoring is reversed for the anxiety absent variables (19 out of 40 items) which broadly assess states of confidence, calmness and security. Total scores range between 20 and 80 for each factor, with higher scores denoting greater anxiety.

Chalder Fatigue Scale (CFS)

Chalder et al. (1993). Severity of fatigue was assessed using the self-reported 11-item CFS. Both physical symptoms (items 1-7; e.g. “Do you have less strength in your muscles?”) and mental symptoms (items 8-11; e.g. “Do you have problems concentrating?”) were evaluated in measuring the severity of overall fatigue. Each item is scored on a 4-level Likert scale i.e. “Less than usual”, “No more than usual”, “More than usual”, “Much more than usual”, with increasing score for each response (0-3). Higher global scores suggest greater fatigue experience.

Childhood Trauma Questionnaire (CTQ)

Bernstein et al. (1994). The 28-item CTQ was used to measure experience of early life adversities across five subscales (emotional abuse, physical abuse, sexual abuse, physical neglect and emotional neglect), and an additional 3-item subscale (minimization and denial) to measure bias in response i.e. tendency for respondent to under-report maltreatment. Higher scores indicate a significant history of childhood trauma.

Perceived Stress Scale (PSS)

Cohen et al. (1983). Perception of global stress was measured using the 10-item PSS. Each item was scored on a 5-point scale (0-5), with scoring reversal for the four reverse-worded items (items 4,5,7,8). Higher scores (ranging from 0 to 40) are indicative of greater stress appraisal.

Life Events Questionnaire (LEQ)

Brugha and Cragg (1990). Evaluation of threatening and stressful life events occurring 6 months preceding assessment) was performed through the 14-item LEQ. The questionnaire is an adapted version of the LTE-Q which appraises threatening events over 12 threatening categories. In this version, a 13th item stressful or major event - “wife or partner gave birth to a child” and a general 14th item – “any other significant event” comprised a binary scale (“yes” or “no”) for each item and a corresponding self-appraised rating to indicate likelihood of the recent life event still affecting the respondent. Number of events was summed to produce LEQ score and corresponding rating were summed to obtain global LEQ rating. Higher prescribed ratings denote acute impact of the occurrence on respondent.

Clinical assessments

Diagnosis of current depressive episode was operationalized by the SCID conducted by trained non-clinician research assistants. The 2-item screening questionnaire for low mood (depression; current 2 weeks) and anhedonia (current 2 weeks) was first administered before further assessment with the full Mood Disorders SCID-I section to ascertain current depressive episode in participants. Given incomplete data on the full SCID-I assessment in some cases, we supplemented the SCID screening outcome (available in all participants) with the HAM-D assessment, also administered by trained research assistants, to index severity of the current depressive episode in cases. We used SCID screening and HAM-D scores on two repeated assessments, at enrolment and immediately prior to MRI, to confirm eligibility criteria were still met at the time of scanning. Previously eligible cases that no longer satisfied SCID screening and HAM-D criteria when re-assessed immediately prior to MRI were excluded from analyses (see **Figure S1**).

Medical comorbidities and concomitant medication

Lifetime history of any serious medical disorder likely to compromise the interpretation of immunological data was exclusionary (including, but not limited to, type 1 and type 2 diabetes, immunological disorders, cardiovascular disorders, malignancies or infection). Similarly, concurrent medication likely to compromise the interpretation of immunological data. e.g. systemic steroids or other anti-inflammatory drugs, was exclusionary.

Potential participants with minor medical comorbidities or concurrent medications were discussed on a case-by-case basis by the study team in light of prior eligibility criteria. Hypothyroidism treated with thyroxine replacement therapy, and with blood concentration of thyroid stimulating hormone in the normal range immediately prior to scanning, was the most frequently included comorbid medical disorder (N=7, 3 in low CRP and 4 in high CRP subgroups). Other comorbid disorders included were: asthma (N=1 in high CRP group); psoriasis (N=1 in low CRP cases); and adrenal insufficiency (N=1 in low CRP group). Six cases (2 in the high CRP and 4 in the low CRP subgroups) were taking antihypertensive medication and three cases (1 in the high CRP and 2 in the low CRP subgroups) were taking statins for hyperlipidaemia. No controls were taking medication for either hypertension or hyperlipidaemia.

Members of the NIMA Consortium

PI = Principal Investigator, EC = Executive committee member

Cambridge. Edward T. Bullmore (PI, EC), Manfred G. Kitzbichler, Junaid Bhatti, Samuel R. Chamberlain, Marta M. Correia, Amber Dickinson, Andy Foster, Clare Knight, Mary-Ellen Lynall, Christina Maurice, Howard Mount, Ciara O’Donnell, Linda J. Pointon, Peter St George Hyslop, Lorinda Turner, Barry Widmer, Guy B. Williams

Cardiff. B. Paul Morgan (PI), Claire Leckey, Angharad Morgan, Caroline O’Hagan, Samuel Touchard

Glasgow. Jonathan Cavanagh (PI, EC), Catherine Deith, John McClean, Alison McColl, Andrew McPherson, Paul Scouller, Murray Sutherland

Independent advisor. H.W.G.M. (Erik) Boddeke (EC)

GSK. Jill Richardson (EC), Shahid Khan, Phil Murphy, Christine Parker, Jai Patel

Janssen. Declan Jones (EC), Peter de Boer, John Kemp, Paul Acton, Wayne C. Drevets, Jeffrey S. Nye (deceased), Gayle Wittenberg, John Isaac, Anindya Bhattacharya, Nick Carruthers, Hartmuth Kolb

Kings College London. Carmine Pariante (PI), Gareth Barker, Heidi Byrom, Diana Cash, Antony Gee, Caitlin Hastings, Nicole Mariani, Anna McLaughlin, Valeria Mondelli, Maria Nettis, Naghmeh Nikkheslat, Karen Randall, Hannah Sheridan, Camilla Simmons, Nisha Singh, Federico Turkheimer, Victoria Van Loo, Marta Vicente Rodriguez, Tobias Wood, Courtney Worrell, Zuzanna Zajkowska

Lundbeck. Niels Plath (EC), Jan Egebjerg, Hans Eriksson, Francois Gastambide, Karen Husted Adams, Ross Jeggo, Christian Thomsen, Jason O'Connor, Jan Torleif Pederson, Brian Campbell, Thomas Möller, Bob Nelson, Stevin Zorn

Oxford. Mary Jane Attenburrow (PI), Alison Baird, Jithen Benjamin, Stuart Clare, Philip Cowen, I-Shu (Dante) Huang, Samuel Hurley, Helen Jones, Simon Lovestone, Francesca Mada, Alejo Nevado-Holgado, Akintayo Oladejo, Elena Ribe, Anviti Vyas

Pfizer. Zoe Hughes (EC), Rita Balice-Gordon, Brendon Binneman, James Duerr, Terence Fullerton, Justin Piro, Tarek Samad, Jonathan Sporn

Southampton. Hugh Perry (PI), Madeleine Cleal, Gemma Fryatt, Diego Gomez-Nicola, Renzo Mancuso

Sussex. Neil Harrison (PI, EC), Mara Cercignani, Charlotte Clarke, Elizabeth Hoskins, Charmaine Kohn, Rosemary Murray, Dominika Wlazly

Bibliography

Beck, A.T., Beamesderfer, A., 1974. Assessment of depression: The depression inventory, in: Psychological measurements in psychopharmacology. S. Karger, Oxford, England, pp. 267–267. doi:10.1159/000395074.

Bernstein, D.P., Fink, L., Handelsman, L., Foote, J., Lovejoy, M., Wenzel, K., et al., 1994. Initial reliability and validity of a new retrospective measure of child abuse and neglect. The American Journal of Psychiatry 151, 1132–1136. doi:10.1176/ajp.151.8.1132.

Brugha, T.S., Cragg, D., 1990. The List of Threatening Experiences: the reliability and validity of a brief life events questionnaire. Acta Psychiatrica Scandinavica 82, 77–81. doi:10.1111/j.1600-0447.1990.tb01360.x.

Chalder, T., Berelowitz, G., Pawlikowska, T., Watts, L., Wessely, S., Wright, D., et al., 1993. Development of a fatigue scale. Journal of Psychosomatic Research 37, 147–153. doi:10.1016/0022-3999(93)90081-P.

Cohen, S., Kamarck, T., Mermelstein, R., 1983. A global measure of perceived stress. Journal of health and social behavior , 385–396.

Cox, R.W., 1996. AFNI: Software for Analysis and Visualization of Functional Magnetic Resonance Neuroimages. Computers and Biomedical Research 29, 162–173. doi:10.1006/cbmr.1996.0014.

First, M.B., Williams, J.B., Karg, R.S., Spitzer, R.L., 2016. SCID-5-CV: structured clinical interview for DSM-5 disorders, clinician version. American Psychiatric Association Publishing.

Fischl, B., 2012. FreeSurfer. NeuroImage 62, 774–781. doi:10.1016/j.neuroimage.2012.01.021.

Fischl, B., Salat, D.H., Busa, E., Albert, M., Dieterich, M., Haselgrove, C., et al., 2002. Whole brain segmentation: automated labeling of neuroanatomical structures in the human brain. Neuron 33, 341–55.

Fornito, A., Zalesky, A., Bullmore, E., 2016. Fundamentals of Brain Network Analysis. Academic Press.

Glasser, M.F., Coalson, T.S., Robinson, E.C., Hacker, C.D., Harwell, J., Yacoub, E., et al., 2016. A multi-modal parcellation of human cerebral cortex. Nature 536, 171–178. doi:10.1038/nature18933.

Hamilton, M., 1960. A Rating Scale for Depression. Journal of Neurology, Neurosurgery & Psychiatry 23, 56–62. doi:10.1136/jnnp.23.1.56.

Knesevich, J.W., Biggs, J.T., Clayton, P.J., Ziegler, V.E., 1977. Validity of the Hamilton Rating Scale for Depression. The British Journal of Psychiatry 131, 49–52. doi:10.1192/bjp.131.1.49.

Kundu, P., Brenowitz, N.D., Voon, V., Worbe, Y., Vértes, P.E., Inati, S.J., et al., 2013. Integrated strategy for improving functional connectivity mapping using multiecho fMRI. Proceedings of the National Academy of Sciences of the United States of America 110, 16187–16192. doi:10.1073/pnas.1301725110.

Kundu, P., Inati, S.J., Evans, J.W., Luh, W.M., Bandettini, P.A., 2012. Differentiating BOLD and non-BOLD signals in fMRI time series using multi-echo EPI. NeuroImage 60, 1759–1770. doi:10.1016/j.neuroimage.2011.12.028.

Poser, B.A., Versluis, M.J., Hoogduin, J.M., Norris, D.G., 2006. BOLD contrast sensitivity enhancement and artifact reduction with multiecho EPI: Parallel-acquired inhomogeneity-desensitized fMRI. Magnetic Resonance in Medicine 55, 1227–1235. doi:10.1002/mrm.20900.

Snaith, R.P., Hamilton, M., Morley, S., Humayan, A., Hargreaves, D., Trigwell, P., 1995. A Scale for the Assessment of Hedonic Tone the Snaith–Hamilton Pleasure Scale. The British Journal of Psychiatry 167, 99–103. doi:10.1192/bjp.167.1.99.

Spielberger, C.D., 1983. Manual for the State-Trait Anxiety Inventory STAI (Form Y) ("Self-Evaluation Questionnaire"). Consulting Psychologists Press .

Wood, T., 2018. QUIT: QUantitative Imaging Tools. doi:10.21105/joss.00656.

Zhang, Y., Brady, M., Smith, S., 2001. Segmentation of brain MR images through a hidden Markov random field model and the expectation-maximization algorithm. IEEE Transactions on Medical Imaging 20, 45–57. doi:10.1109/42.906424.

List of Tables

S1 Detailed participant inclusion and exclusion criteria . 6
S2 Socio-demographic and clinical data on the quality controlled micro-structural data. 7
S3 KS-test for all qMT measures. 7
S4 Areas affected by CRP. 8
S5 Sub-cortical regions whose cortical connections are correlated with CRP. 9

List of Figures

S1 Flow diagram outlining progression of participants through to analyses. 10
S2 Distributions of the different qMT metrics per group. . 11
S3 Functional connectivity strength by group. 12
S4 Sensitivity analysis 13
S5 Sensitivity analysis combined 14
S6 Comparison to main results 14
S7 ICA analysis 15
S8 PD and FC map overlap 16
S9 Potential confounds from motion 16
S10 Continuous depression scales 17
S11 PD effect of continous depression scale 17
S12 Controls only CRP correlation 18
S13 Controls only mediation analysis 18
S14 Scan site effect 19
S15 Pairwise correlations of behavior metrics 19
S16 FC vs phenotype 20

Tables

Table S1: **Detailed participant inclusion and exclusion criteria** Related to section Study design and sample.

Group	Inclusion Criteria	Exclusion Criteria
**All participants**	• Able and willing to provide informed consent<br>• Proficiency in English (spoken, written and comprehension)<br>• Aged 25-50 years (inclusive)<br>• Body mass index (BMI) < 36 kg/m2<br>• Able and willing to fast for 8 hours prior to blood specimen collection<br>• Willing to abstain from strenuous exercise for 72 hours prior to assessment	• Lifetime history or comorbid medical disorders that are likely to compromise immune profile (including, but not limited to, immunological disorders, cardiovascular disorders, malignancies, acute and chronic infections)<br>• Medication (in healthy controls) and concomitant therapeutics (non-antidepressant in clinical respondents) that may alter immune profile (including, but not limited to, corticosteroids)<br>• Active substance abuse or dependence within the last 6 months prior to screening<br>• Contraindications to MRI<br>• Pregnancy or breastfeeding<br>• Participation in clinical drug trial within the last 12 months prior to screening
**Healthy controls**		• Current or past history of major psychiatric disorder as defined by the DSM-5<br>• Treatment with a monoaminergic antidepressant for depressive symptoms or any other indication<br>• Baseline serum/plasma high-sensitivity CRP > 3mg/L
**Cases**	• Meet the DSM-5 screening criteria for major depressive disorder (MDD) operationalized by the Structured Clinical Interview for DSM-5 (SCID)<br>• Score at least 13 (from first 17 items) on the observer-rated Hamilton Rating Scale for Depression (HAM-D)<br>• Baseline serum/plasma high-sensitivity CRP < 3mg/L for “lo-CRP Cases” or “non-inflamed depression” arm<br>• Baseline serum/plasma high-sensitivity CRP > 3mg/L for “hi-CRP Cases” or “inflamed depression” arm	• Lifetime history of bipolar disorder or other non-affective psychotic disorders

Table S2: **Socio-demographic and clinical data on the quality controlled micro-structural data.** Related to section Sample.

	Controls	Cases	p	loCRP Cases	hiCRP Cases	p
**n**	37	73		44	29	
**sex (female/male)**	21/16	50/23	0.292	24/20	26/3	0.002
**age, years**	35.7 (7.4)	37.2 (7.2)	0.321	36.4 (6.9)	38.3 (7.8)	0.290
**body mass index**	24.6 (4.0)	26.6 (3.8)	0.014	25.3 (3.1)	28.7 (3.9)	<0.001
**C-reactive protein, mg/L**	0.9 (0.7)	3.0 (3.0)	<0.001	1.0 (0.7)	6.0 (2.6)	<0.001
**clinician-rated depression**	0.4 (0.7)	19.7 (5.4)	<0.001	19.9 (5.7)	19.4 (4.9)	0.736
**self-rated depression**	1.7 (2.6)	26.2 (9.6)	<0.001	25.8 (9.8)	26.8 (9.3)	0.691
**state anxiety**	27.0 (8.2)	51.2 (10.2)	<0.001	51.8 (10.0)	50.1 (10.7)	0.491
**trait anxiety**	29.2 (6.1)	60.8 (9.7)	<0.001	60.8 (10.3)	60.9 (9.1)	0.977
**fatigue**	10.9 (2.0)	20.5 (5.7)	<0.001	20.6 (5.5)	20.3 (6.1)	0.839
**anhedonia**	0.2 (0.7)	5.2 (3.6)	<0.001	5.8 (3.6)	4.3 (3.4)	0.076
**childhood trauma**	38.8 (5.4)	54.2 (15.6)	<0.001	57.3 (16.7)	49.6 (12.5)	0.037

Table S3: **KS-test for all qMT measures.** (Bonferroni corrected)

sequence	group comparison	method	alternative	statistic	p	p (Bonferroni)
k_{bf}	high CRP Cases and Controls	Two-sample KS test	two-sided	0.096	0.064	1
	high CRP and low CRP Cases	Two-sample KS test	two-sided	0.14	0.0011	**0.033**
f_b	high CRP Cases and Controls	Two-sample KS test	two-sided	0.08	0.18	1
	high CRP and low CRP Cases	Two-sample KS test	two-sided	0.14	0.0011	**0.033**
$T2_f$	high CRP Cases and Controls	Two-sample KS test	two-sided	0.15	0.0005	**0.015**
	high CRP and low CRP Cases	Two-sample KS test	two-sided	0.14	0.002	0.06
$T2_b$	high CRP Cases and Controls	Two-sample KS test	two-sided	0.072	0.29	1
	high CRP and low CRP Cases	Two-sample KS test	two-sided	0.085	0.13	1
$T1_f$	high CRP Cases and Controls	Two-sample KS test	two-sided	0.074	0.25	1
	high CRP and low CRP Cases	Two-sample KS test	two-sided	0.11	0.023	1
**PD**	**high CRP Cases and Controls**	Two-sample KS test	two-sided	0.18	$\mathbf{P < 9.1 \times 10^{-6}}$	$\mathbf{P < 2.7 \times 10^{-4}}$
	**high CRP and low CRP Cases**	Two-sample KS test	two-sided	0.2	$\mathbf{P < 6.4 \times 10^{-7}}$	$\mathbf{P < 1.9 \times 10^{-5}}$

Table S4: **Areas affected by CRP.** Related to Proton density: between-group differences and correlation with CRP and Depression-related changes in functional connectivity.

Areas which showed significant positive (red) or negative (blue) correlation between PD and CRP.

Area	Template code	Template name	Yeo network
**Dorsolateral prefrontal**	9a	Area 9 anterior	DMN
**Posterior cingulate**	RSC	Retro Splenial Complex	DMN
	PCV	PreCuneus Visual Area	DMN
	7m	Area 7m	DMN
	POS1	Parieto-Occipital Sulcus Area 1	DMN
	v23ab	Area ventral 23 a+b	DMN
	d23ab	Area dorsal 23 a+b	DMN
	31pv	Area 31p ventral	DMN
	31pd	Area 31pd	DMN
	31a	Area 31a	DMN
	ProS	ProStriate Area	VIS
**Ventral stream visual cortex**	VMV1	Ventro Medial Visual Area 1	VIS
**Inferior frontal**	IFJa	Area IFJa	FP
	IFSa	Area IFSa	FP
	p47r	Area posterior 47r	FP
**Orbital and polar frontal**	p10p	Area posterior 10p	FP
	a10p	Area anterior 10p	FP
	OFC	Orbital Frontal Complex	LIM
**Anterior cingulate and medial prefrontal cortex**	pOFC	posterior OFC Complex	LIM
	25	Area 25	LIM
**Premotor cortex**	6r	Rostral Area 6	VA
**Sensori-motor associated paracentral cortex**	5m	Area 5m	MOT

Areas which showed significant group differences in functional connectivity (FC).

Area	Template code	Template name	Yeo module
**anterior cingulate and medial prefrontal cortex**	a24	Area a24	DMN
	d32	Area dorsal 32	DMN
	p32	Area p32	DMN
	10r	Area 10r	DMN
	9m	Area 9 Middle	DMN
	s32	Area s32	DMN
	p24	Area posterior 24	DMN
	a32pr	Area anterior 32 prime	FP
	25	Area 25	LIM
	33pr	Area 33 prime	VA
	a24pr	Anterior 24 prime	VA
	p32pr	Area p32 prime	VA
**association auditory cortex**	STGa	Area STGa	DMN
	STSda	Area STSd anterior	DMN
	STSvp	Area STSv posterior	DMN
	STSva	Area STSv anterior	DMN
**dorsal stream**	V6A	Area V6A	VIS
**dorsolateral prefrontal cortex**	8BL	Area 8B Lateral	DMN
	9p	Area 9 Posterior	DMN
	9a	Area 9 anterior	DMN
**early auditory cortex**	52	Area 52	MOT
	MBelt	Medial Belt Complex	MOT
**inferior parietal cortex**	PGi	Area PGi	DMN
**insular and frontal opercular cortex**	AAIC	Anterior Agranular Insula Complex	DMN
**lateral temporal cortex**	TE1a	Area TE1 anterior	DMN
**orbital and polar frontal cortex**	47m	Area 47m	DMN
	10d	Area 10d	DMN
**posterior cingulate cortex**	RSC	RetroSplenial Complex	DMN
	PCV	PreCuneus Visual Area	DMN
	7m	Area 7m	DMN
	POS1	Parieto-Occipital Sulcus Area 1	DMN
	v23ab	Area ventral 23 a+b	DMN
	d23ab	Area dorsal 23 a+b	DMN
	31pv	Area 31p ventral	DMN
	31pd	Area 31pd	DMN
	ProS	ProStriate Area	VIS
	DVT	Dorsal Transitional Visual Area	VIS
**ventral stream**	VMV2	VentroMedial Visual Area 2	VIS
**sub-cortex**	Hippocampus	Hippocampus	sub

Table S5: **Sub-cortical regions whose cortical connections are correlated with CRP.** Related to section Functional connectivity: correlation with CRP and mediation analysis.

Putamen-cortical connections negatively correlated with CRP in depressed cases.

Area	Template code	Template name	Yeo network	"New"	PD ~ CRP
**Dorsolateral prefrontal**	8Av	area 8 av	FP	yes	
	8Ad	area 8 ad	DMN	yes	
	i6-8	inferior 6-8 transitional area	FP	yes	
**Posterior cingulate**	7m	area 7m	DMN	no	positive $R = 0.34$, $P < 0.001$
	d23ab	area dorsal 23 ab	DMN	no	positive $R = 0.29$, $P < 0.01$
**Inferior parietal**	IP1	area intraparietal 1	FP	no	
	PGs	area PGs	DMN	no	
**Superior parietal**	7Pm	medial area 7p	FP	yes	
	7Pl	lateral area 7p	DA	yes	

Thalamo-cortical connections negatively correlated with CRP in depressed cases.

Area	Template code	Template name	Yeo network	"New"	PD ~ CRP
**Dorsolateral prefrontal**	46	area 46	FP	no	negative $R = 0.24$, $P < 0.05$
	i6-8	inferior 6-8 transitional area	FP	yes	
**Inferior parietal**	IP2	area intraparietal 2	FP	no	
**Premotor cortex**	6r	Rostral Area 6	VA	no	negative $R = 0.29$, $P < 0.01$

Hippocampus-cortical connections correlated with CRP in depressed cases.

Area	Template code	Template name	Yeo network	PD ~ CRP
**posterior cingulate**	POS1	Parieto-Occipital Sulcus Area 1	DMN	positive $R = 0.37$, $P < 0.001$
	v23ab	Area ventral 23 a+b	DMN	positive $R = 0.41$, $P < 0.001$
**anterior cingulate and medial prefrontal**	10r	Area 10r	DMN	
**dorsolateral prefrontal**	9-46d	Area 9-46d	FP	negative $R = 0.27$, $P < 0.01$

Figures

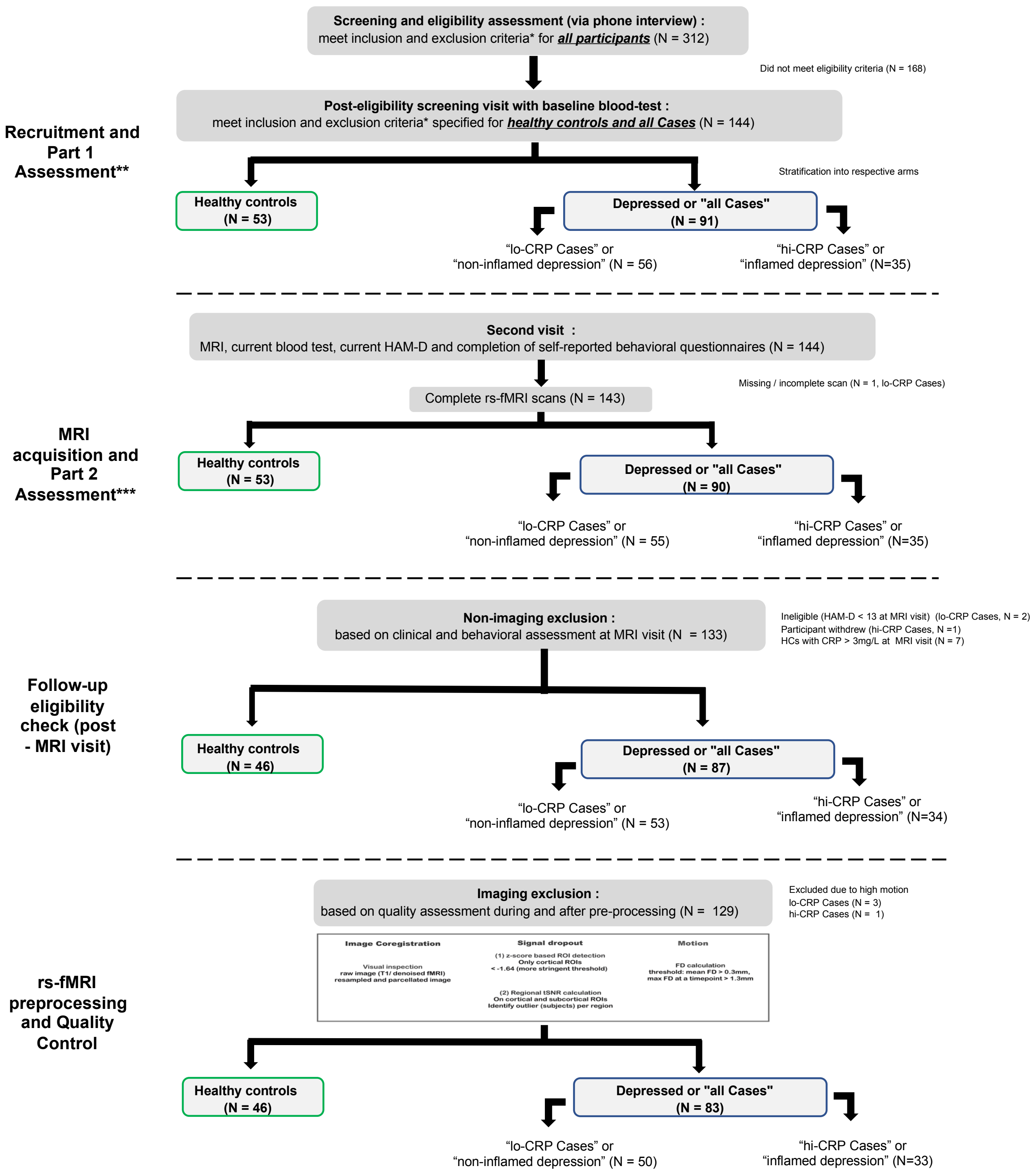

Figure S1: **Flow diagram outlining progression of participants through to analyses.** Related to section Study design and sample.

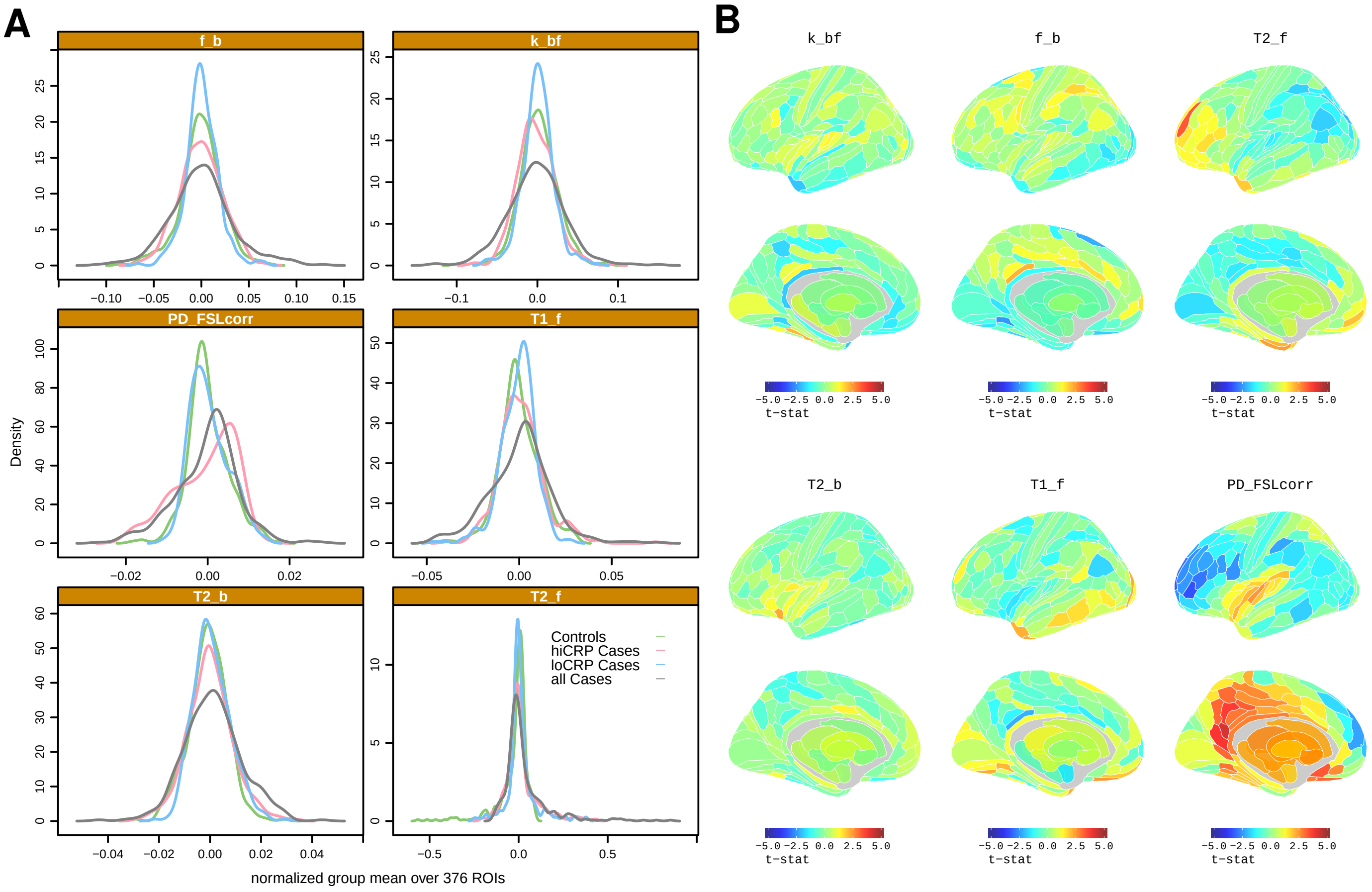

Figure S2: **Distributions of the different qMT metrics per group.** (**A**) Distributions per group of the six different qMT metrics. (**B**) Map of the CRP dependence of the six different qMT metrics per region. Both, for group as well as CRP dependence, the PD metrics shows the largest effect.Related to section Proton density: between-group differences and correlation with CRP.

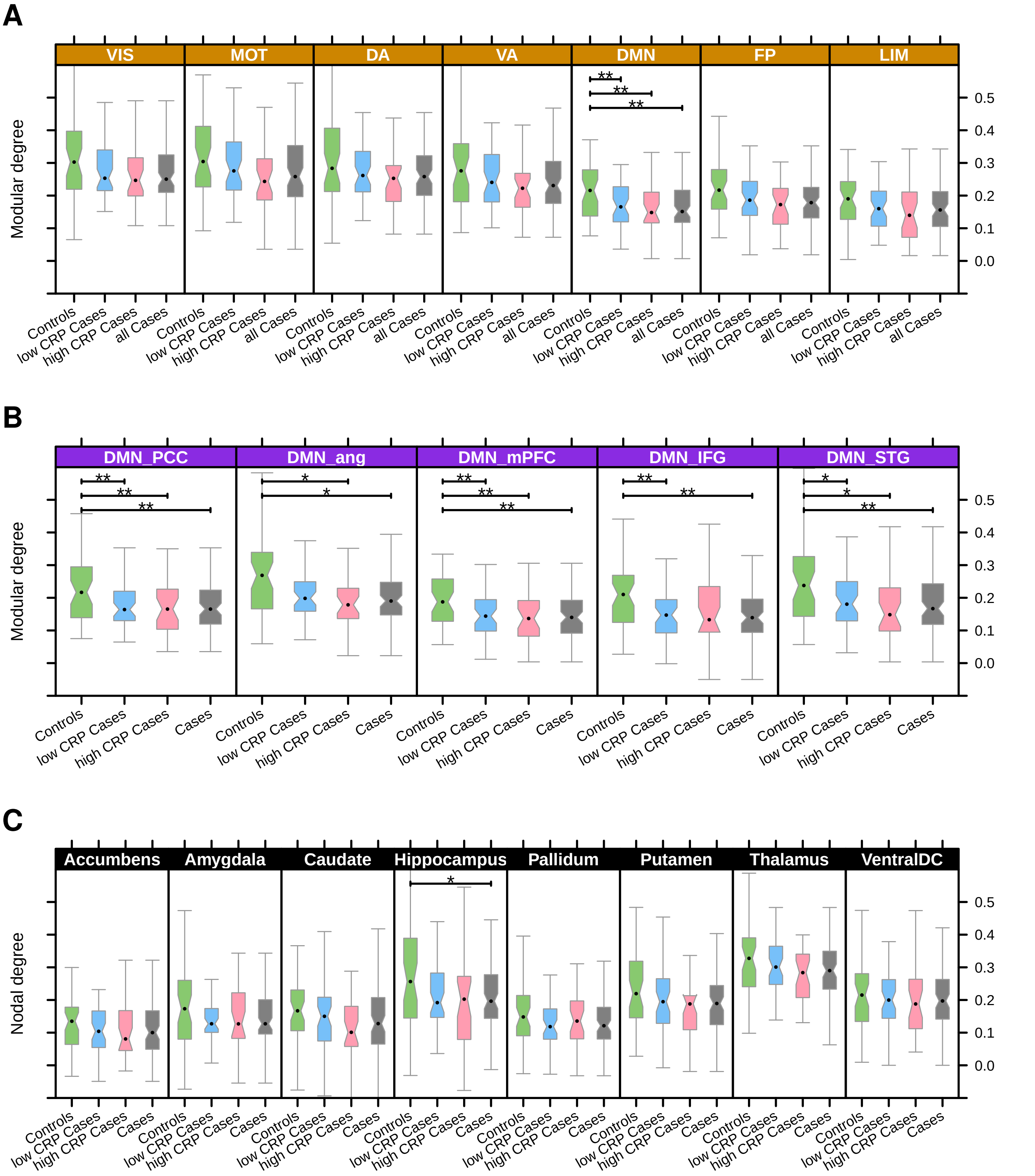

Figure S3: **Functional connectivity strength by group.** Related to section Functional connectivity: depression-related differences. (**A**) Nodal connectivity strength per group per Yeo modules (**B**) Expanded view of DMN panel in A, showing DMN sub-modules. Here and above ** means p<0.01 uncorrected (**C**) Nodal connectivity strength per group per sub-cortical regions

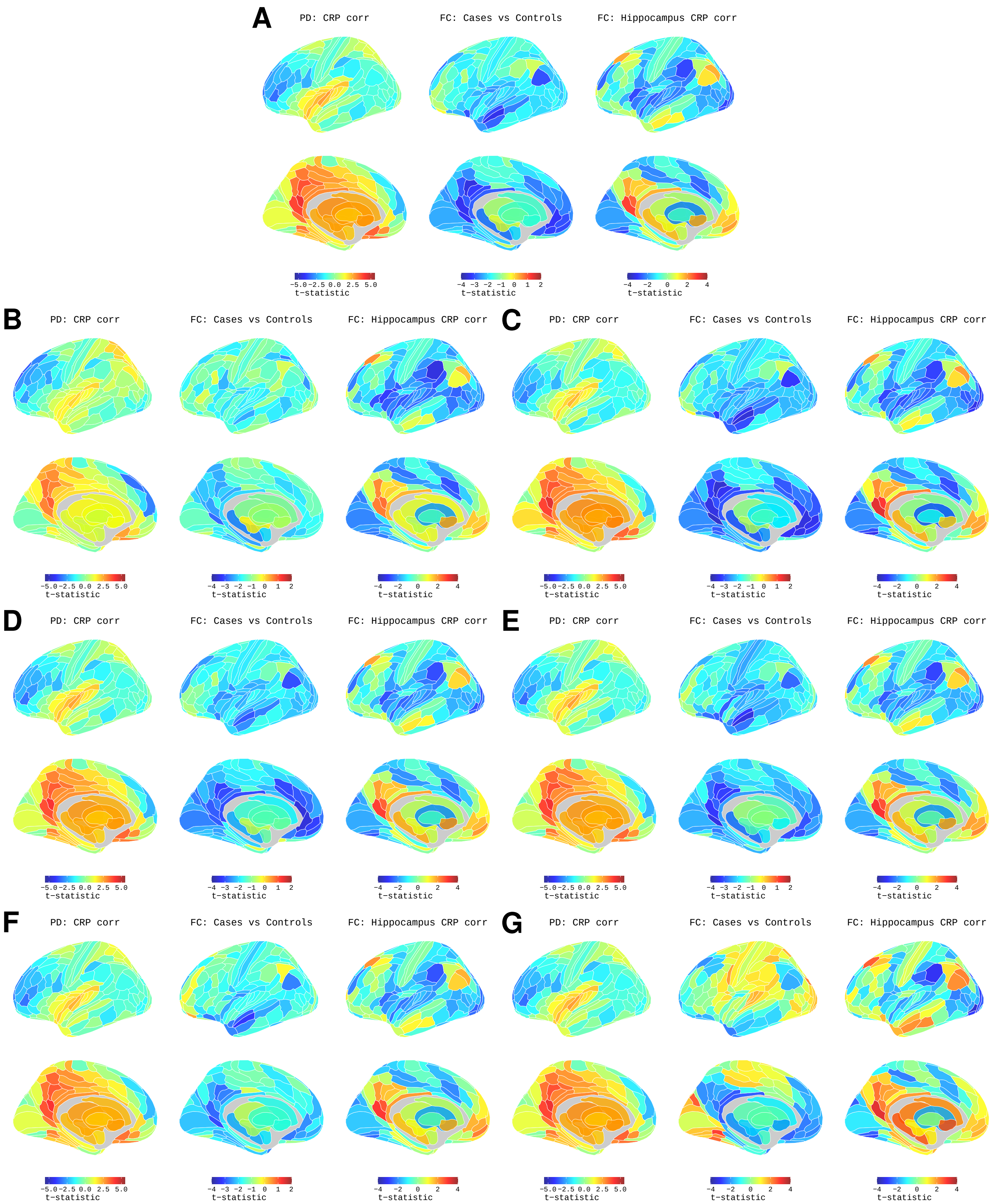

Figure S4: **Sensitivity analysis** Related to section Methodological issues and limitations. First panel at the top (**A**): baseline results as presented in the main paper; from left to right: cortical maps of CRP correlation in PD, case-control difference in FC, and CRP correlation in the Hippocampus connectivity. Below, for comparison: same as (**A**), but taking into account Sex (**B**), body mass index (BMI, **C**), childhood adversity (CTQ, **D**), total number of antidepressants (**E**), whether currently on antidepressants (**F**), and Global Signal Regression (**G**).

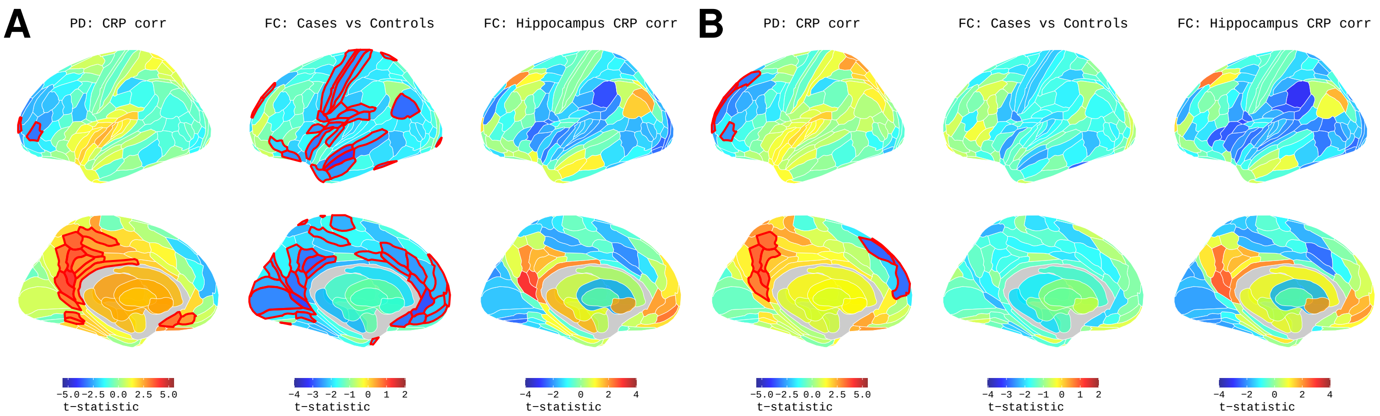

Figure S5: **Sensitivity analysis combined** Analoguous to previous **Figure S4**, but (a) combining all nuisance regressors (childhood adversity, total number of antidepressants, whether currently on antidepressants, but not body mass index) in one model and (b) once more the same all inclusive model for females only

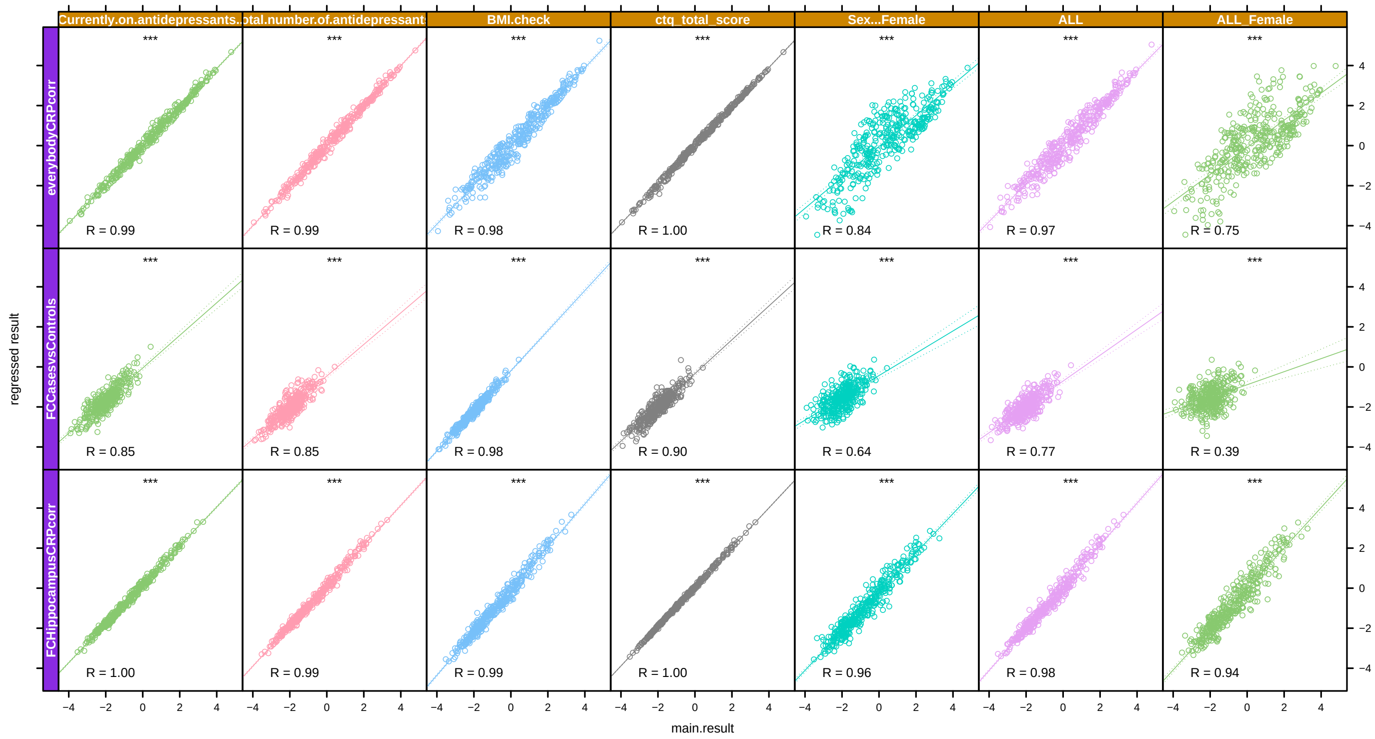

Figure S6: **Comparison to main results** Numerical comparison of t-stat results for the different nuisance regressor models (y-axis) vs the results from the main paper (x-axis)

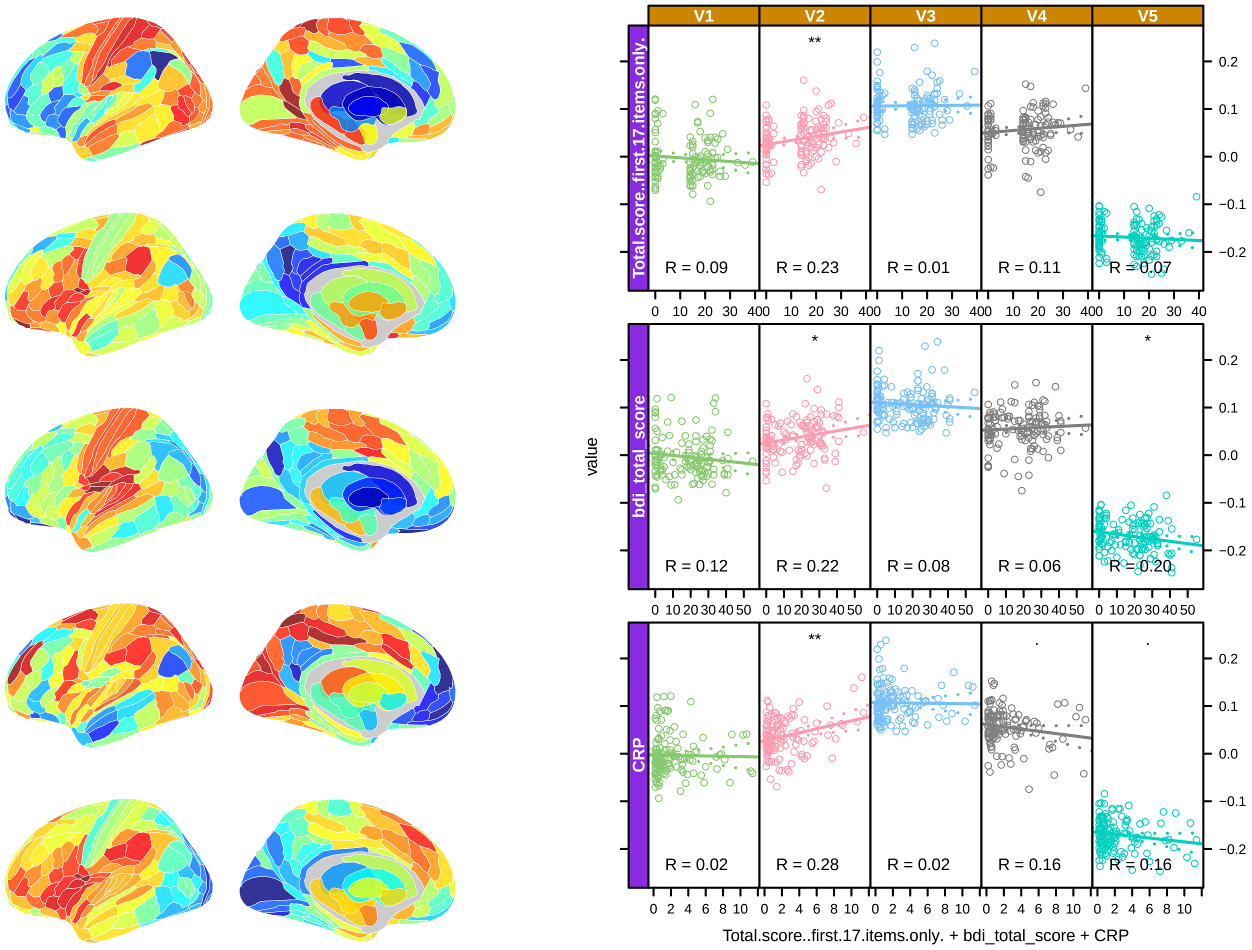

Figure S7: **ICA analysis** ICA analysis using 5 components as an alternative approach to using Yeo networks. Component V2 (second map from top) corresponds roughly to the DMN and also shows the strongest dependence on depression (using a continuous scale, first two rows of scarrerplots on the right) and CRP (third row on the right). This is consistent with the pre-defined Yeo network based analysis presented in the main paper.

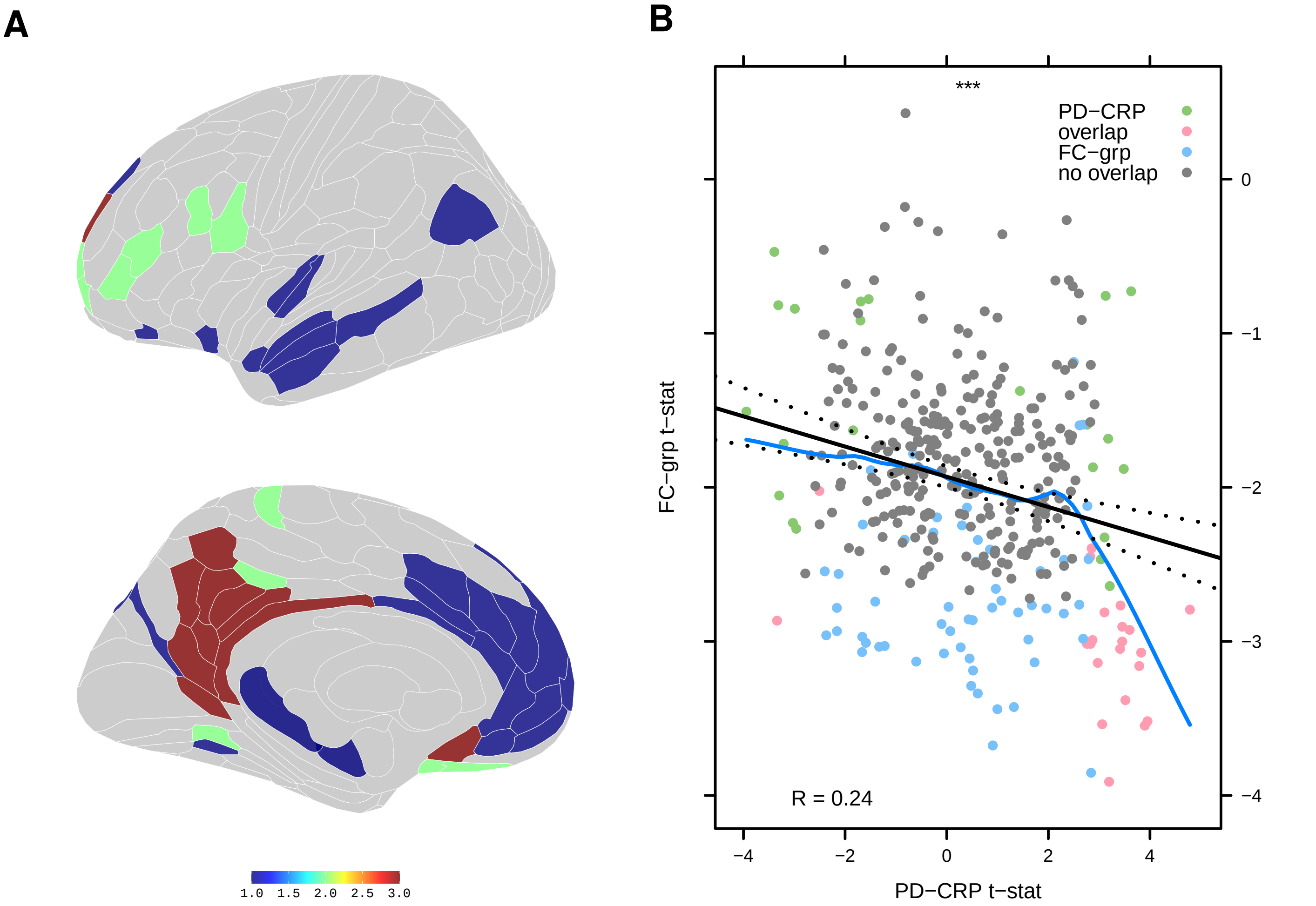

Figure S8: **PD and FC map overlap** (a) Overlap of significant PD (CRP effect) and FC (group effect) regions. (b) PD effect vs FC effect (t-stats) across regions. Color codes relate to overlap colors on the right (but are not the same; gray: no overlap, red: only PD, blue: only FC, green: overlap of both)

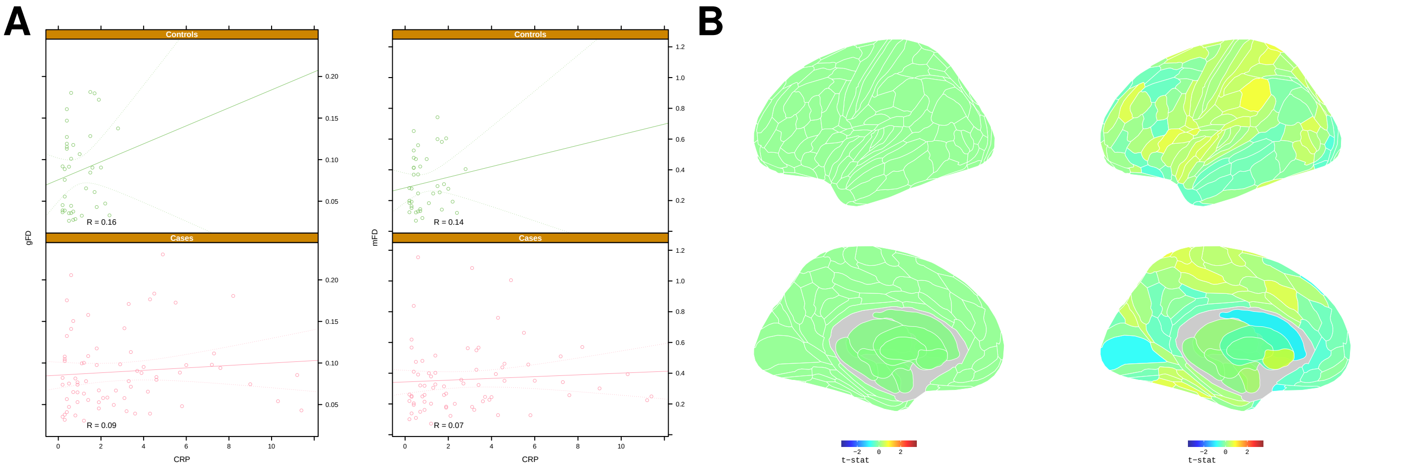

Figure S9: **Potential confounds from motion** (a) Dependence of motion on CRP. (b) Absence of any significant correlation between motion and functional connectivity (FC). gFD: mean global framewise displacement, mFD: maximum FD over the whole length of the scan.

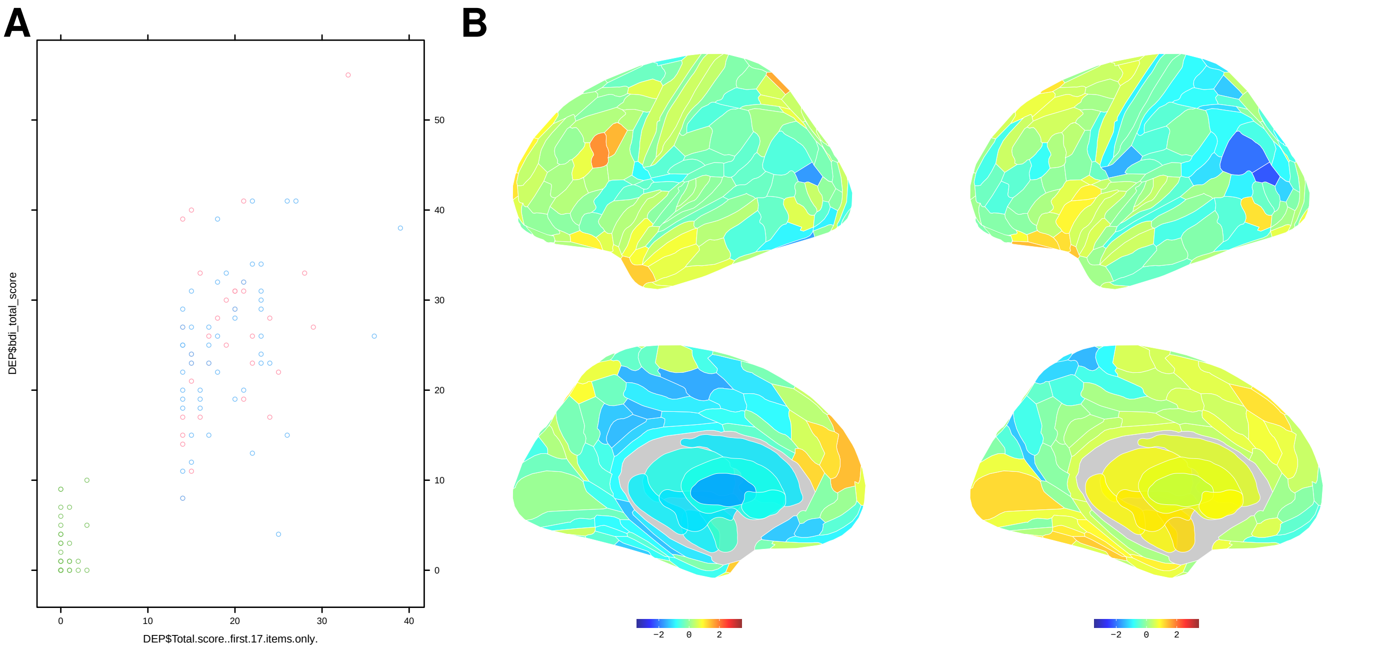

Figure S10: **Continuous depression scales** (a) Comparison of HAM-D and BDI. (b) No significant effects of continuous depression scales on PD (consistent with no patient effect in analysis prsented in the main paper).

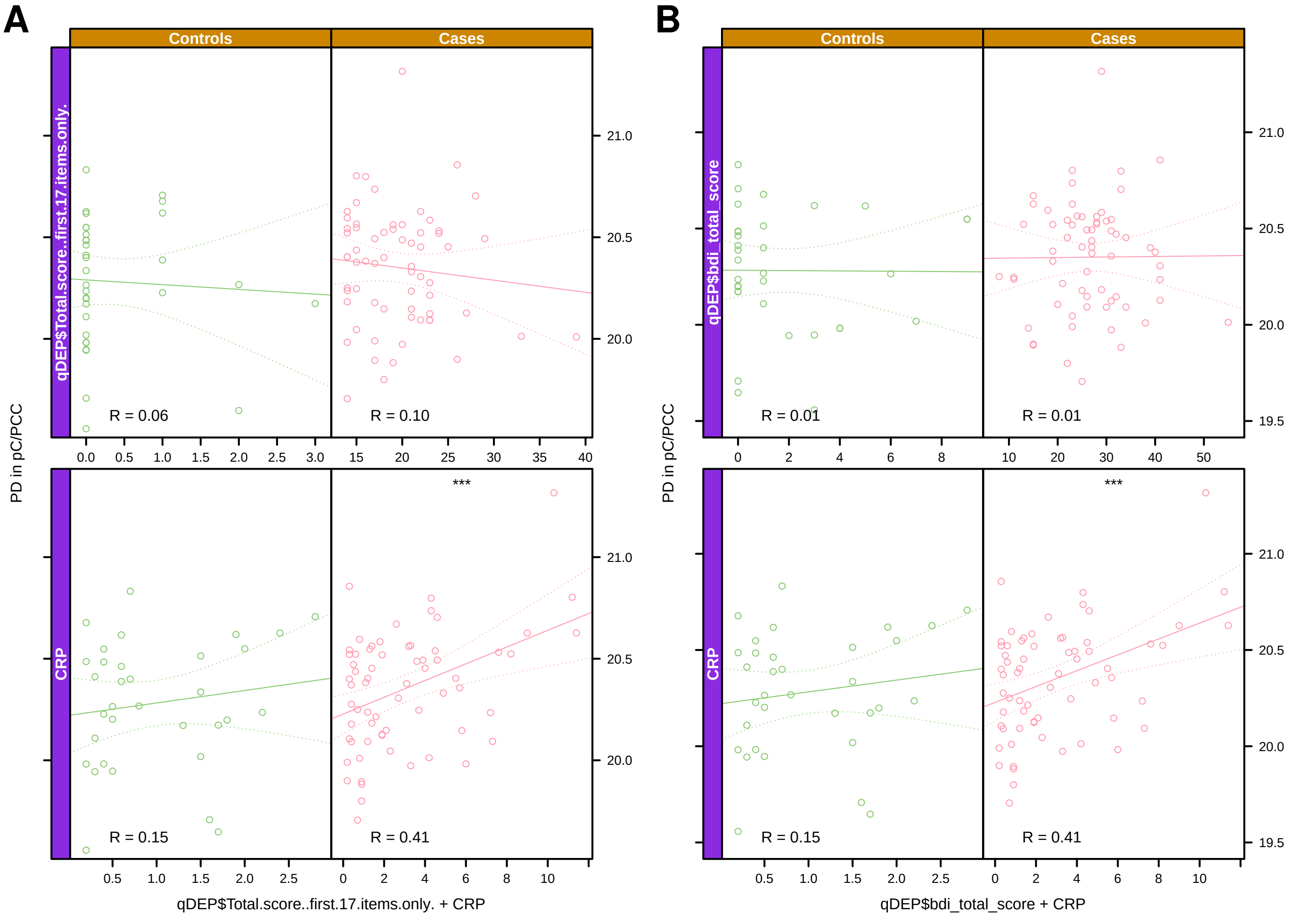

Figure S11: **PD effect of continous depression scale** Numerical comparison of PD effects on continuous depression scales vs effect of CRP. (a) HAMD total score, (b) BDI score. Please note that x-axis scales vary between control and patient groups.

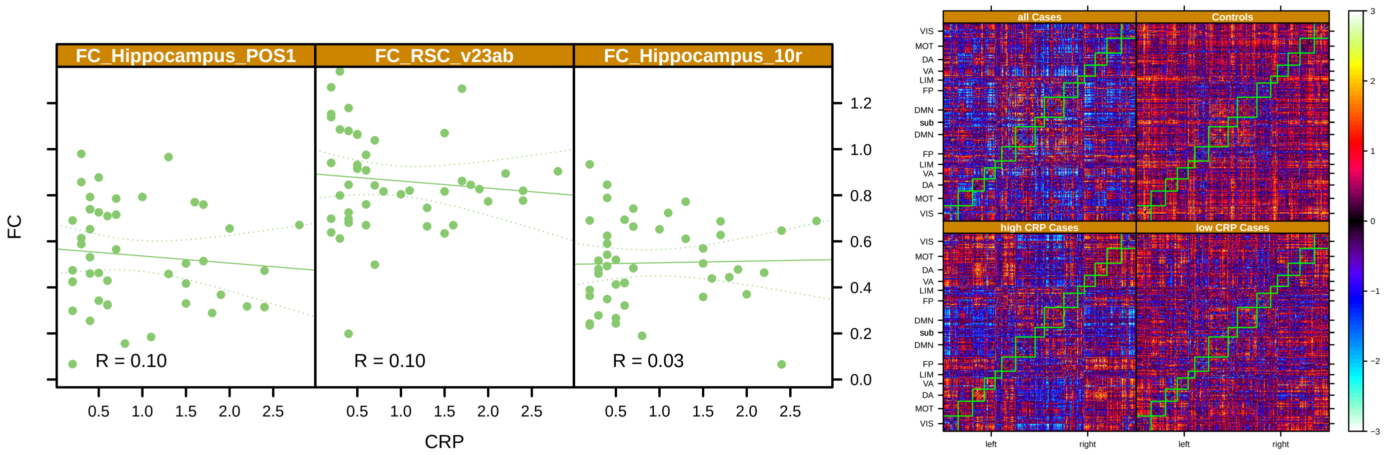

Figure S12: **Controls only CRP correlation** Left: For the three connections shown in the main paper, relationship between connectivity and CRP for healthy controls only. Right: Correlation (Fisher z-transformed) of functional connectivity with CRP for each pair of regions as a matrix, shown separately for each group of subjects.

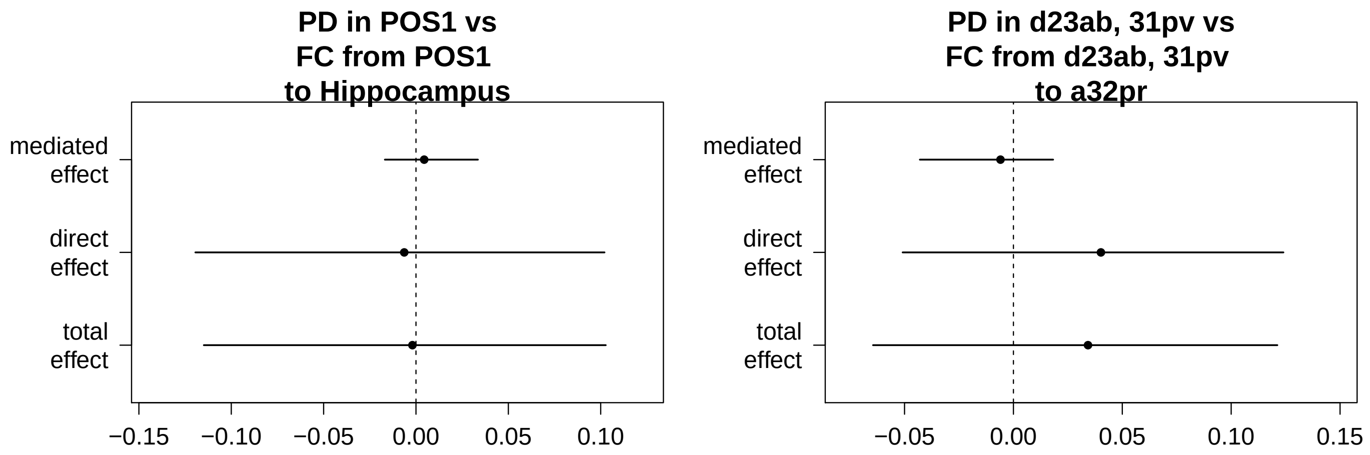

Figure S13: **Controls only mediation analysis** For comparison in controls only: mediation analysis of the direct effect of CRP on PD in posterior cingulate cortical area POS1 (left) and posterior cingulate cortical areas d23ab and 31pv (right). Compare with Fig 3B and 3C in the submitted paper.

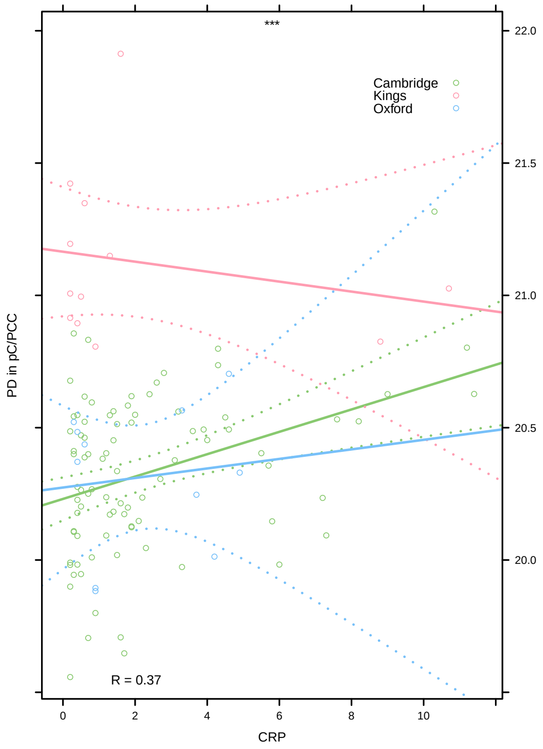

Figure S14: **Scan site effect** PD-CRP effect by scan site. In the PD results presented in the submitted manuscript KCL has been excluded since during pre-processing the quantitative MRI measurements could not be brought into alignment between different scanner manufacturers (Siemens vs GE). This figure demonstrates post-hoc that including scans from KCL would not have resulted in more statistical power.

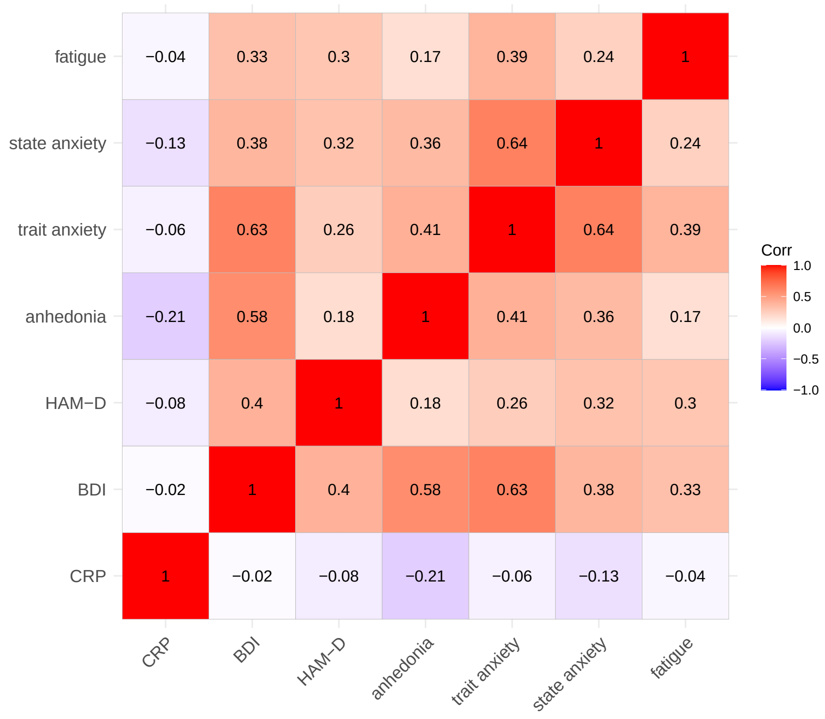

Figure S15: **Pairwise correlations of behavior metrics**

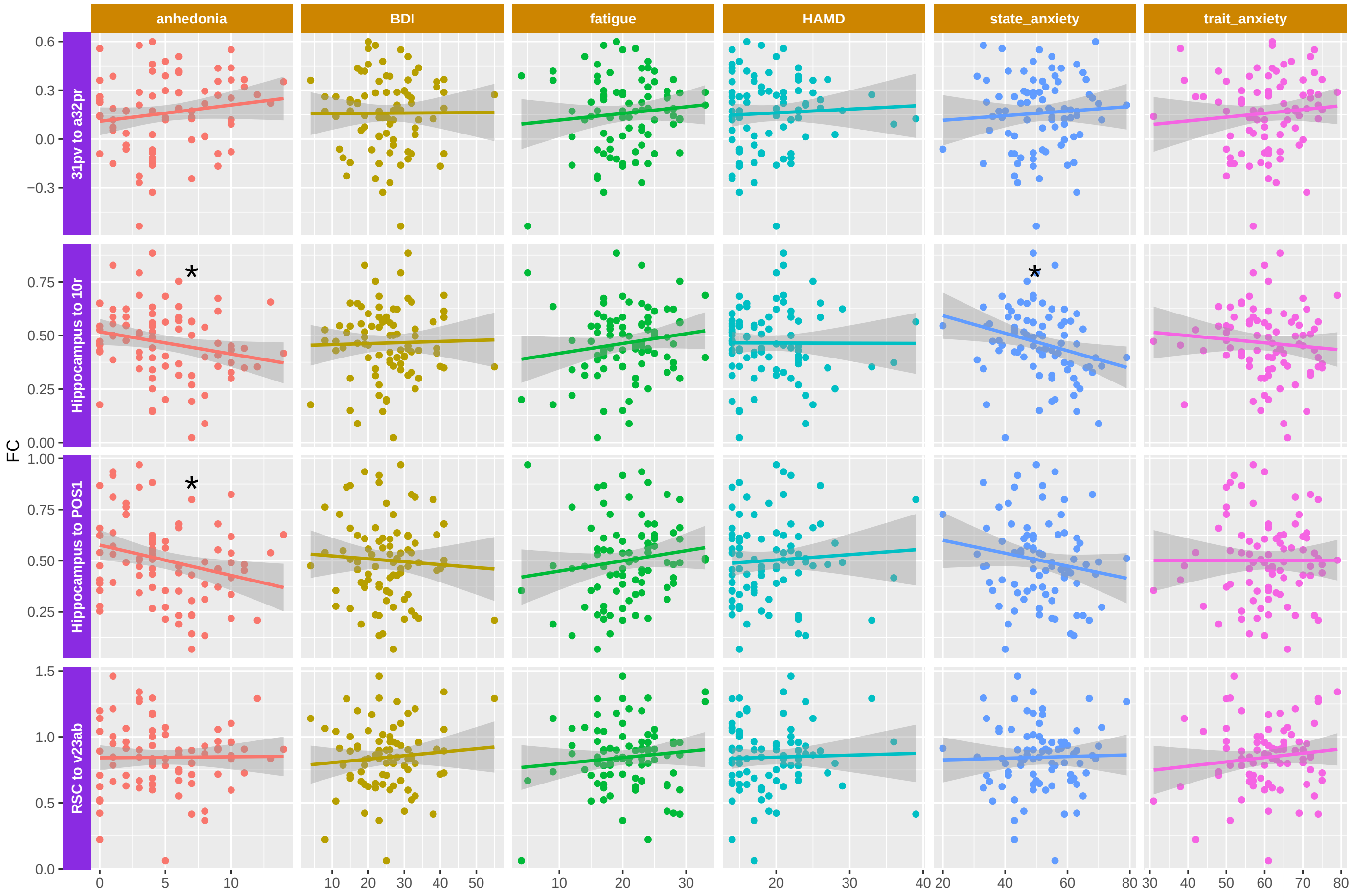

Figure S16: **FC vs phenotype** Dependence on various behavioural metrics of functional connectivity strength in 4 pairwise connections (top to bottom) that showed significant modulation by CRP (see **Figure 3** in the main paper). We did not observe any significant scaling of phenotypic features with the discrete CRP-sensitive functional connections (* indicates P < 0.05 uncorrected).
